# INTerest of electrophysiological and functional EXploration in the evaluation of symptomatic impact of superior semicircular canal DEHIscence syndrome (INTEX-DEHI study): Study protocol for a reliability and validity study

**DOI:** 10.1371/journal.pone.0331763

**Published:** 2025-09-18

**Authors:** Quentin Legois, Fabrice Giraudet, Yohan Gallois, Benoit Lepage, Eugen Ionescu, Jean-François Vellin, Valérie Franco-Vidal, Akil Kaderbay, Marie-Jeanne Vellin, French Association of Superior Semicircular Canal Dehiscence syndrome, Mathieu Marx

**Affiliations:** 1 ORL, Otoneurology and ORL pediatric department, CHU Toulouse, Toulouse, France; 2 UMR5549, CNRS, Brain and Cognition Research Center, Toulouse, France; 3 UMR1107, INSERM, Neurosensory Biophysics Team, Clermont-Ferrand, France; 4 Department of Medical Genetics, University Hospital of Clermont-Ferrand, France; 5 SoluSons Hearing Center, Clermont-Ferrand, France; 6 UR4156, Neuropsycholinguistics Laboratory, Toulouse, France; 7 Methodological Research Support Unit, Faculty of Medicine, University of Toulouse, Toulouse, France; 8 Department of Audiology and Otoneurological Explorations, Civil Hospitals of Lyon, Lyon, France; 9 Department ORL, Saint-Vincent Clinic, Saint-Denis (La Réunion), France; 10 Oto-Rhino-Laryngology-Head and Neck Surgery Department Bordeaux, Hôpital Pellegrin, Bordeaux, France; 11 Oto-Rhino-Laryngology-Head and Neck Surgery Department, Montpellier University, CHU Gui de Chauliac, Montpellier, France; PLOS: Public Library of Science, UNITED KINGDOM OF GREAT BRITAIN AND NORTHERN IRELAND

## Abstract

**Background:**

Interest in Superior Semicircular Canal Dehiscence (SSCD) has increased, but its diagnosis and management remain challenging. Despite advances in imaging and electrophysiological tests, many patients face long diagnostic delays and significant distress due to unclear symptom severity. While some patients show no symptoms despite radiological evidence, others experience disabling effects. This research aims to bridge the gap between objective tests and symptom severity, improving the understanding and management of SSCD.

**Methods:**

This is a prospective, non-randomized, longitudinal observational clinical study including repeated measures. It is part of the inter-regional hospital research program “INTEX-DEHI”, involving five university hospitals. Patients diagnosed with unilateral superior semicircular canal dehiscence via CT scan will be followed over four weeks with three visits spaced 15 days apart. The main objective is to describe the clinical and paraclinical characteristics of SSCD and analyze their correlation. Electrophysiological, functional, and clinical measurements will be collected at each visit.

**Discussion:**

The relationship between electrophysiological tests and the symptomatic impact of superior semicircular canal dehiscence remains underexplored. This research aims to bridge this gap by studying both common and specific VEMP frequencies, as well as EcoG and WBT, to assess their sensitivity, specificity, and diagnostic value. While this study proposes innovative tools to objectively assess symptoms, challenges remain, particularly the underdiagnosis of the syndrome and selection biases, especially in patients with fluctuating symptoms. Additionally, the diversity of clinical presentations and the absence of symptoms in some patients complicate the interpretation of results.

**Trial registration:**

Clinicaltrials.gov, NCT06170398.

## Introduction

Interests on superior semicircular canal dehiscence (SSCD) has been increasing in recent years, associated with the exponential number of publications [1]. Although knowledge of the syndrome is increasingly well-established within the ENT community, the value of electrophysiological and functional investigations in this context remains poorly defined. We still observe many patients experiencing prolonged therapeutic wandering before a diagnosis is made. Some of them suffer, develop a significant psychological distress, whether due to the prolonged uncertainty of this wandering or the severity of the symptoms they must endure [2]. This wandering is all the more regrettable given that SSCD represents one of the main treatable causes of vertigo, tinnitus, and hyperacusis [3].

The symptomatic presentation of patients remains highly heterogeneous, both in the type of symptoms exhibited and the severity of their impact [4,5]. However, a consensus has been reached to define the diagnostic criteria for this variant [6] of third mobile window (TMW) syndrome [7]. These criteria include the presence of at least one symptom characteristic of a “third mobile window” in the inner ear, such as bone conduction hyperacusis, sound- or pressure-induced vertigo or oscillopsia, or pulsatile tinnitus. Diagnostic signs or specific tests are required, including characteristic nystagmus provoked by sound stimuli or pressure changes, audiometry showing negative low-frequency bone conduction thresholds, or enhanced VEMP responses. Additionally, high-resolution CT imaging of the temporal bone with multiplanar reconstruction is essential to observe SSCD. Finally, these symptoms and signs must be clearly distinguishable from other vestibular disorders.

Atypical symptoms are attributed to SSCD, which behaves like a third window in the otic capsule, in addition to the round and oval windows, whose presence is physiological [8]. This results in a disruption of the biomechanics of the labyrinthine fluids by reducing the impedance within the vestibular system [9–11].

The heterogeneity of symptoms is further compounded by significant variability in their impact, with some patients presenting with radiological SSCD without symptoms, while others are severely disabled, thus creating a true continuum in the severity of the symptomatic presentation. Considering the radiological prevalence of SSCD, estimated between 0.8% and 3% according to studies [12,13], and the cadaveric prevalence estimated at 0.5% [14], it is likely that most individuals with SSCD are paucisymptomatic or asymptomatic.

In cases of radiological doubt, the use of Vestibular Myogenic Evoked Potentials (VEMP) can help confirm or strengthen the suspicion of SSCD diagnosis [15]. Acoustic stimulation (clicks or tone bursts) of the otolithic organs results in a change in sternocleidomastoid muscle (cervical VEMP) activity due to saccular activation, as well as a change in inferior oblique muscle (ocular VEMP) activity induced by utricular activation [16–18]. Approximately 13 ms after sound stimulation, an inhibition of contraction is observed in the ipsilateral sternocleidomastoid muscle, while activation of the contralateral inferior oblique muscle occurs around 10 ms after stimulation [16–18]. In patients with SSCD, the acoustic stimulation thresholds required to trigger a saccular response are generally lower, while the amplitudes of the utricular response are higher [19–21]. However, no consensus has yet been reached regarding the reduction in stimulation thresholds or the amplification of amplitude. The cVEMP technique shows a sensitivity of 84% and a specificity of 80%, indicating a strong ability to identify superior semicircular canal dehiscence (SSCD) [22]. Furthermore, the amplitude of air-conducted oVEMPs demonstrates particularly high diagnostic accuracy for SCDS, with a sensitivity of 100% and a specificity of 89% at a threshold of 16.7 µV [20]. The 2000 Hz tone burst frequency for cVEMPs [19] and the 4000 Hz tone burst frequency for oVEMPs [23] are specific frequencies at which only dehiscent ears would generate responses.

Electrocochleography (EcoG) and Wideband tympanometry (WBT) are two common electrophysiological tests that recently have been evaluated in the assessment of patients with SSCD. The first test provides cochlear and nerve responses to an auditory stimulus from an electrode positioned in the ear canal or invasively on the cochlea [24,25]. EcoG shows promising performance in the diagnosis and follow-up of SSCD, with a reported sensitivity of 89% and a specificity of 70% [22]. The SP/AP ratio is significantly higher in patients with SSCD, exhibiting a sensitivity of 92.3% and a specificity of 94.0% [26]. The second is a functional test that measures the acoustic energy absorbed by the tympano-ossicular system using a tympanometry probe inserted into the ear canal, delivering a wideband frequency sound as a click [27].

These two additional tests, which may assist in the diagnosis and monitoring of SSCD, have been the subject of limited research. EcoG would reveal a high summating potential (SP) to action potential (AP) ratio in the context of SSCD compared to non-dehiscent ears, with the SP/AP ratio normalizing after surgery [22,26,28–30]. WBT could highlight a peak in absorbance in the middle and inner ear around 1 kHz [31,32].

However, these diagnostic tools have not demonstrated utility in the objective assessment of symptomatic impact. The absence of an objective marker for symptom severity makes therapeutic decision-making particularly challenging, relying solely on the reported functional complaints. To our knowledge, no study to date has examined the relationship between objective functional assessment results (obtained by imaging and electrophysiology) and the subjective symptomatic impact of the syndrome. Such a marker could prove essential in guiding therapeutic direction once the diagnosis is made. This research thus aims to establish a link between the objective abnormalities, whether electrophysiological or radiological, and the severity of symptoms observed in SSCD.

## Materials and methods

### Study design/settings

This is a prospective, non-randomized, observational, multicenter, longitudinal clinical study. It is part of an inter-regional hospital clinical research program named “INTEX-DEHI”, which involves five university hospitals. This study will focus on a cohort of patients with unilateral SSCD diagnosed via CT scan. Its objective will be to describe the clinical and paraclinical characteristics of the syndrome and their correlation. Patients included in the study will be followed in the ENT departments of the five university hospitals. Patients will be seen three times over a period of four weeks (± 5 days) in order to assess the test-retest reliability of the measurements. One investigator per center will be responsible for enrolling patients into the study, while only two experimenters will be tasked with administering the protocol (one for the four centers, and another for the remote center).

The study began on April 29, 2024, is still ongoing, and is planned for a duration of 24 months. Patient recruitment will therefore be completed on April 29, 2026, and data collection will be completed on May 29, 2026 (one month after the last inclusion). The data will then be processed to extract the results, which will be available within three months of that date.

### Participants and selection criteria

Participants are gradually included in the study as they arrive in the ENT departments for their first consultation, if they wish to do so, and if they meet the study’s selection criteria. For convenience and to increase the statistical power of our study, patients with strictly unilateral dehiscence will be selected in order to have a control condition consisting of the healthy ears of the patients in our cohort.

Subjects are eligible to participate if they meet the following inclusion criteria: [1] have strictly unilateral dehiscence diagnosed by high-resolution CT scan (0.5 mm slices, reconstruction in the Pöschl and Stenver planes [33–35]) of the temporal bone, without necessary criteria for symptomatic involvement, [2] if present, the symptoms suggestive of SSCD must be stable, in the context of diagnostic follow-up and monitoring, [3] be an adult patient (18 years or older) able to read and understand French, [4] be affiliated with or a beneficiary of a social security system, and [5] provide free, informed, and signed consent (no later than the day of inclusion).

Patients are excluded if they meet any of the following criteria: [1] have bilateral SSCD diagnosed by high-resolution CT scan of the temporal bone, in order to avoid uncertainty in attributing the symptoms to one, the other, or both SSCDs, [2] have a doubtful diagnosis of SSCD on high-resolution CT scan of the temporal bone (uncertainty about the loss of bone continuity, technical characteristics of the scan, absence of specific reconstructions in the superior canal plane and its perpendicular plane), [3] have an associated otological or otoneurological pathology that could have a symptomatic impact comparable to that of SSCD (chronic otitis media, Eustachian tube dysfunction, endolymphatic hydrops, otosclerosis, and other ossicular anomalies with a normal eardrum, definite Meniere’s disease, vestibular migraine), [4] are under legal guardianship or another protection regime (guardianship, curatorship), and [5] are pregnant.

### Primary outcomes

To achieve the primary objective, which is to determine whether there is a correlation between the intensity of symptoms generated by SSCD and the results collected from electrophysiological and functional testing, a set of results will need to be collected. First, the severity of the symptoms experienced by the patients will be recorded. To do this, we translated the Naert questionnaire [5] using Beaton’s method [36], with the author’s consent, which lists all the symptoms (31 symptoms) that patients with SSCD may experience [4]. The severity of these symptoms is measured using a 5-point Likert scale (no problem, mild problem, moderate problem, severe problem, problem as severe as possible).

Secondly, electrophysiological and functional cVEMP and oVEMP, EcoG and WBT data will be recorded (Eclipse® electrophysiological device and Titan® tympanometer, Interacoustics).

For cVEMP and oVEMP, we will collect the thresholds (in dBnHL), latencies (in ms), and amplitudes (in µV) of the evoked potentials for common 500 Hz tone burst frequency stimulus [37–46], as well as for atypical 2 kHz tone-burst stimulus for cVEMP [19] and 4 kHz tone-burst stimulus for oVEMP [23], which are thought to be specific to SSCD.

For electrocochleography, 85 dBnHL clicks will be used and the amplitude (in µV) and latency (in ms) of the SP and AP, SP/AP amplitude ratio and area under the curve will be collected [22,26,28–30].

Regarding the WBT, the resonance frequency as well as the absorbance percentage at 1kHz will be acquiered [32].

The precise stimulation parameters are listed in S1 Appendix.

### Secondary outcomes

To analyze the impact of SSCD on quality of life, the EQ-5D-5L scale (mobility, self-care, pain, discomfort/anxiety, depression [47]) will be administered, and the intensity of the 4 cardinal symptoms (pulsatile tinnitus, autophony, vertigo induced by loud sound, and change of pressure) of the syndrome using a Visual Analog Scale (VAS) rated from 0 to 10 will be assessed (0 for the absence of symptoms and 10 for extremely disabling symptoms).

The Naert questionnaire will be administered (alongside the other subjective scales) to patients during 3 separate visits to assess the validity, reliability, and inter-visit reproducibility of the results.

For each visit, the presence or absence of a Valsalva-induced nystagmus [6] using videonystagmoscopy will be assessed; this will allow us to analyze the reliability and reproducibility of this clinical sign.

As for the electrophysiological and functional measures, they will also be repeated during 3 separate visits, as well as twice per visit to assess intra-visit reproducibility.

Finally, by measuring the length, width, and surface area of the dehiscence on high-resolution CT scan, defining its location on the canal (anterior arm, arch, posterior arm), and identifying the anatomical structure in which the dehiscence opens (superior petrous sinus, meninges), we will examine whether there is a correlation with the clinical and paraclinical measurements mentioned above [35]. We will do the same with each patient’s baseline tonal audiometry.

### Participation timelines

**Fig 1:**
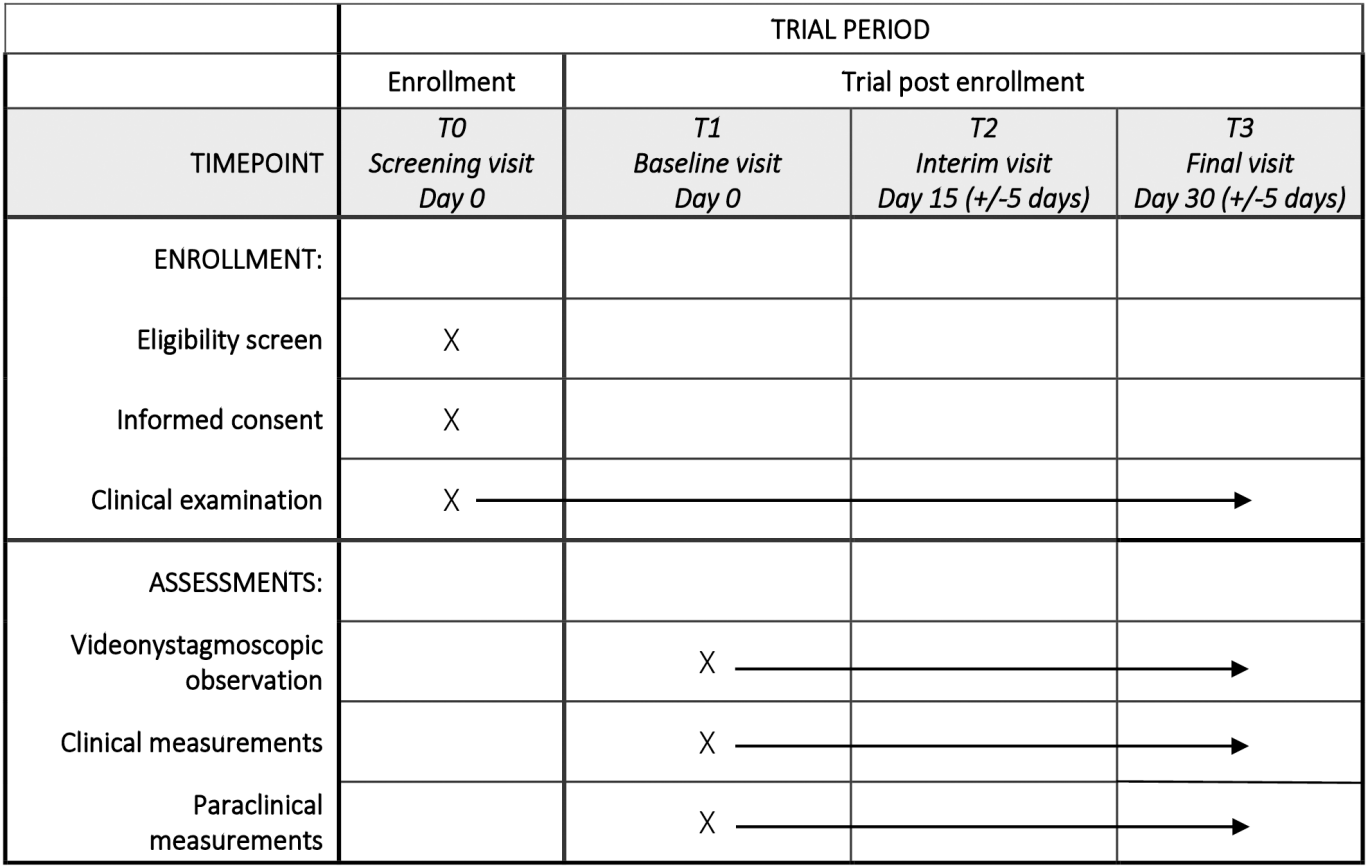
SPIRIT schedule of enrollment and assessments.

Fig 1 presents the participant timeline, detailing the schedule of enrollment and assessments throughout the study.

During the pre-inclusion visit, the temporal bone CT scan previously performed will be reviewed by the investigator to formally confirm the diagnosis of SSCD. A clinical examination, including bilateral otoscopy, is also performed. Next, the investigator will inform the participant and answers all their questions regarding the objective, the nature of the constraints, the foreseeable risks, and the expected benefits of the research. A copy of the information sheet and the consent form will be provided to the participant by the investigator.

Once the patient will be included in the study by the investigator, three visits will be scheduled, each 15 days apart (+/- 5 days). During each visit, the patient will be seen by an experimenter who will ensure the complete administration of the protocol (clinical and paraclinical measurements). To reduce bias, only two experimenters (one for the four centers, and another for the remote center) will perform all functional and electrophysiologic explorations with the same equipment, the same stimulation and the same acquisition parameters. Both experimenters will conduct all measurements for their respective patients. The paraclinical measurements will be performed using standardized equipment that remains consistent across visits.

**Fig 2:**
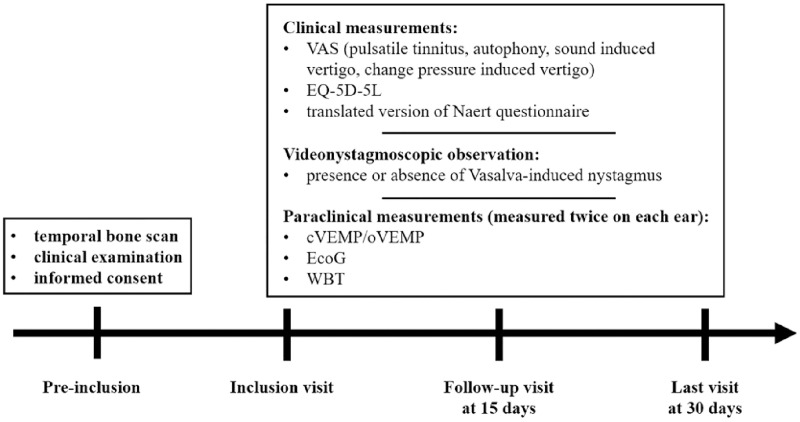
Flowchart of the INTEX-DEHI protocol, and the measurements taken at each visit.

During each visit, the VAS scores for each cardinal symptoms (pulsatile tinnitus, autophony, sound-induced vertigo, pressure change-induced vertigo), the EQ-5D-5L, and the translated version of the Naert questionnaire will be recorded by experimenters. The presence of a Valsalva-induced nystagmus will then be observed by the experimenter using videonystagmoscopy, and the electrophysiological (cVEMP/oVEMP, EcoG) and functional (WBT) explorations will be proceeded with. Two measurements on each ear will be performed (healthy and dehiscent). The participation timeline is presented in the flowchart in Fig 2.

### Recruitment

Participants will be recruited during their ENT consultation visits. The ENT correspondents of each center will be informed about the study and may refer potentially eligible patients to their respective referring center, where the investigator for the study is located. Recruitment may also be facilitated by the neuroradiology teams associated with each center, who are involved in diagnosing SSCD through radiological means and can inform the relevant patients about the study. Each of the 5 participating centers is a national or international Neurotologic expert center for functional testing of hearing and balance. As stated in the informed consent form, patients may withdraw from participation in the research protocol without the need to justify their decision.

### Sample size

The reliability of the electrophysiological indicators will be assessed by estimating intra-class correlation coefficients (ICC). Assuming ICCs > 0.80 and using the 6 repeated measurements of each parameter, 100 participants are needed to estimate ICCs with 95% confidence intervals (CI) of width less than 0.10 (which we consider to be sufficiently precise). Assuming that 10% to 13% of patients might be loss to follow-up before the last visit, the total sample size is 115 patients.

### Data collection

A series of data are collected during our protocol, but an exhaustive list would have been too long, so we report them in S2 Appendix. These independent variables are encoded in an electronic Case Report Form (eCRF). Access to the data will be limited, as it will be restricted to the clinical research associate and the principal investigator. The data set will be validated by the data-manager and analyzed by the statistician.

### Statistical method

The data will be globally described, outlier values will be corrected whenever possible, and missing data will be recorded. A flowchart will illustrate patient follow-up, including inclusions and premature exits with their reasons.

Statistical tests will be used to assess the reliability and validity of the electrophysiological, clinical, and Naert questionnaire measurements.

The reliability of repeated measurements will be analyzed using the Bland and Altman graphical method, which estimates the systematic bias and 95% limits of agreement, as well as intra-class correlation coefficients (ICCs) to evaluate intra-patient (6 measurements), intra-visit (2 measurements), and inter-visit (3 visits) reliability. If inter-visit reliability is sufficient, convergent and divergent validity will be assessed by analyzing correlations between electrophysiological indicators and symptoms (presence, intensity, Naert questionnaire), distinguishing between measurements from the affected and unaffected sides.

A multivariate unsupervised descriptive analysis including principal component analysis and clustering will be performed to identify patient profiles. A supervised modeling approach using partial least squares regression may be considered to predict symptomatology based on electrophysiological and anatomical indicators.

The stability of the measurements will be tested using a repeated-measures ANOVA (if normality is met) or the Friedman test (if normality is not met), while the agreement between measurements within the same visit will be evaluated using Bland and Altman analysis. Differences between measurements will be assessed using a paired t-test (if normality is met) or the Wilcoxon signed-ranks (if normality is not met). If a significant effect is found using ANOVA, post-hoc comparisons will be performed using the Tukey HSD test.

The psychometric properties of the Naert questionnaire will be analyzed through inter-item correlations, Cronbach’s alpha coefficient, and factorial analysis. Its test-retest reproducibility will be evaluated using ICC and Bland and Altman analysis.

We will calculate the sensitivity, specificity, positive predictive value, and negative predictive value for each assessment to evaluate their ability to detect the presence of SSCD. A ROC curve analysis will also be conducted to assess the overall performance of each test and determine the optimal discrimination threshold. The significance level will be set at 0.05.

The data will be globally described, outlier values will be corrected whenever possible, and missing data will be recorded. A flowchart will illustrate patient follow-up, including inclusions and premature exits with their reasons.

Statistical analyses will be performed using Rstudio (version 2025.05.1 + 513).

### Research ethics and safety assessment

The electrophysiological and functional tests planned for this research are non-invasive procedures commonly used, even daily, in neurotologic consultations. However, they may sometimes cause discomfort due to the presence of a probe in the external auditory canal, electrodes attached to the skin of the neck (cervical VEMP) or cheekbone (ocular VEMP), or brief sounds presented at high intensity. No additional adverse effects are expected. This research protocol is subject to the regulations of the Jardé law, Research Involving the Human Person – category 2 (low-intervention, “low risk”), and has been approved by the Ouest I ethics committee (SI number: 23.02993.000220/ national number: 2023-A01856-39). The promoter of this study is the Toulouse University Hospital.

## Discussion

A recent bibliometric analysis conducted by Patel *et al.* shows that among the 100 most cited articles on superior semicircular canal dehiscence, none have explored the link between electrophysiological/functional tests and the symptomatic impact of the syndrome [48]. As a result, therapeutic decision-making largely depends on the impact of symptoms, an aspect often overlooked in research.

For VEMP, it is likely that our results will confirm those of numerous previous studies regarding the common 500 Hz tone burst frequency. However, we are also interested in specific frequencies which is an innovative approach, as only isolated studies have been conducted on these (2 kHz tone-burst stimulus for cVEMP and 4 kHz tone-burst stimulus for oVEMP). We will analyze their specificity and sensitivity. This would allow us to offer an effective routine electrophysiological test for the diagnosis and follow-up of SSCD syndrome. Additionally, there are very few studies on EcoG and WBT in relation to SSCD, which is why we also hope to identify electrophysiological and functional markers through these explorations that could objectively assess the symptomatic impact of the syndrome in patients.

This research protocol will allow us to highlight useful tools (questionnaires, electrophysiological tests, functional tests) for objectively assessing patients’ symptoms, while also enabling us to study the quality of these measurements (validity, reliability, reproducibility).

However, we will face some challenges, as although this syndrome and his variants are frequently observed anatomically, it is most likely underdiagnosed, which could introduce a bias in patient inclusion. Our goal is to cover a wide range of clinical presentations, from asymptomatic patients to those with severe symptoms. It is likely that we will miss some asymptomatic patients, which is necessary to complete our large cohort. On the other hand, it is also very rare to have patients with the full clinical picture (autophony, pulsatile tinnitus, vertigo triggered by loud sounds and pressure changes). We will therefore take this into account and be cautious when interpreting and extrapolating our results. We also have a selection bias, as by only selecting patients with stable symptoms (without any scheduled surgical treatment in the next 3 months), we are not including patients with highly fluctuating symptoms, who are potentially the most disabled.

The advantage of using high-resolution CT scans for all included patients is that it will allow us to examine the correlation between the presence or absence of a radiological dehiscence and the presence or absence of vestibular and auditory symptoms, although this has already been studied in the literature [13]. By measuring the size of the dehiscence on the high-resolution CT scan [12], we will be able to explore its relationship with symptomatic impact, as assessed by the Naert scale, as well as with the results of electrophysiological and functional tests.

We are also limited because the dehiscences are sometimes bilateral. We chose to exclude patients in whom SSCD was bilaterally present, as it was the best way for us to have a control condition and be able to compare the healthy ear and the dehiscent ear of each included patient.

We have ensured that the study is multicentric in order to cover as much territory as possible. This approach will increase the likelihood of recruiting a broader symptomatic panel, and we will also have a better chance of recruiting the 100 subjects we aim for.

The study follows to the Standard Protocol Items: Recommendations for Interventional Trials (SPIRIT) statement [49]. The SPIRIT checklist is provided as S1 File.

## Trial status

The latest version of this protocol, as presented above, is version 2.1, established on September 29, 2023. The study inclusions are scheduled over a period of 24 months starting from the first inclusion, with each center operating independently (therefore, the project end date varies from one center to another). The first inclusion took place on April 29, 2024 at the Toulouse center. To date, 10 patients have been seen at this center. Regarding the other centers, 7 patients are scheduled at the CHU of Saint-Denis (La Réunion), and 13 patients at the CHU of Lyon. Currently, no patients are scheduled at the CHU centers of Montpellier and Bordeaux, although the research project is active locally.

## Supporting information

S1 Appendix Stimulation parameters. (PDF)

S2 Appendix Data collected. (PDF)

S1 File SPIRIT checklist. (PDF)

S2 File INTEX-DEHI english version. (DOCX)
