# Supplementary material for: INTerest of electrophysiological and functional EXploration in the evaluation of symptomatic impact of superior semicircular canal DEHIscence syndrome (INTEX-DEHI study): Study protocol for a reliability and validity study
Source: PLoS One. 2025 Sep 18;20(9):e0331763. doi: 10.1371/journal.pone.0331763 (PMC12445554; doi:10.1371/journal.pone.0331763)
Supplement: S1 Appendix — (PDF) [file pone.0331763.s001.pdf]

WBT Stimulation Parameters:

- +200 daPa / -400 daPa

EcoG Stimulation Parameters:

- Stimulus: Click
- Rate: 7.7/sec
- Polarity: Alternating / Rarefaction / Condensation
- Intensity: 85 dB
- Gradually increase intensity
- Low-pass filter at amplifier input: 5000 Hz
- High-pass filter at amplifier input: 3.3 Hz, 6 dB/oct
- Preliminary low-pass filter settings: 3000 Hz
- Preliminary high-pass filter settings: None
- Response curve volt/div: 20 nanovolt
- Stimulation technique: Insert
- Number of acquisitions: 1000
- Recording window: from -2 ms to 8 ms
- Artifact rejection: ±40 microvolt (92 dB)
- Wave reproducibility: from 0.0 to 3.0 ms

(No chewing gum, patient far from PC and eclipse unit, eyes closed, relaxed, no movement, proper preparation, remove watch, ground connection at the table)

cVEMP Stimulation Parameters:

- Stimulus: Tone Burst (4 ms rise, 4 ms plateau, 4 ms fall)
- Frequency: Two frequencies will be tested—500 Hz (used in clinical practice) and 2000 Hz (specific to SSCD, NOIJ K., 2018)
- Rate: 5.1/sec
- Polarity: Rarefaction
- Intensity: See above and increase gradually
- Low-pass filter at amplifier input: 1000 Hz
- High-pass filter at amplifier input: 10 Hz, 6 dB/oct

- Preliminary low-pass filter settings: 200 Hz
- Preliminary high-pass filter settings: None
- Response curve volt/div: 20 microvolt
- Stimulation technique: Headphone
- Number of acquisitions: 200
- Recording window: from -20.0 ms to 60.0 ms
- Artifact rejection: ±800 microvolt (66 dB)
- Wave reproducibility: from 0.0 to 30.0 ms
- EMG: 18.0 – 36.0 – 147.2 – 180.0 microvolt RMS

oVEMP Stimulation Parameters:

- Stimulus: Tone Burst (4 ms rise, 4 ms plateau, 4 ms fall)
- Frequency: Two frequencies will be tested—500 Hz (used in clinical practice) and 4000 Hz (specific to SSCD, MANZARI L., 2013)
- Rate: 5.1/sec
- Polarity: Rarefaction
- Intensity: See above and increase gradually
- Low-pass filter at amplifier input: 1000 Hz
- High-pass filter at amplifier input: 10 Hz, 6 dB/oct
- Preliminary low-pass filter settings: 200 Hz
- Preliminary high-pass filter settings: None
- Response curve volt/div: 5 microvolt
- Stimulation technique: Headphone
- Number of acquisitions: 200
- Recording window: from -20.0 ms to 60.0 ms
- Artifact rejection: ±400 microvolt (72 dB)
- Wave reproducibility: from 0.0 to 30.0 ms
- EMG: 3.0 – 3.8 – 24.0 – 30.0 microvolt RMS
