# Supplementary material for: INTerest of electrophysiological and functional EXploration in the evaluation of symptomatic impact of superior semicircular canal DEHIscence syndrome (INTEX-DEHI study): Study protocol for a reliability and validity study
Source: PLoS One. 2025 Sep 18;20(9):e0331763. doi: 10.1371/journal.pone.0331763 (PMC12445554; doi:10.1371/journal.pone.0331763)
Supplement: S2 Appendix — (PDF) [file pone.0331763.s002.pdf]

Variables recorded during WBT (for each trace):

- Resonance frequency
- % absorbance at 1000 Hz

Variables recorded during EcoG (for each trace):

- Baseline start
- Baseline end
- SP peak
- AP1
- AP peak
- SP amplitude
- AP amplitude
- SP/AP ratio

Variables recorded during VEMP (for each trace):

For cVEMP:

- P13 latency
- N23 latency
- P13 amplitude
- N23 amplitude
- Peak-to-peak latency
- Peak-to-peak amplitude
- RMS (Root Mean Square)
- EMG standard deviation
- Wave reproducibility
- Artifact rejection
- Rosengren formula (2015): Peak-to-peak amplitude / RMS
- Asymmetry ratio = (Larger amplitude – Smaller amplitude) / (Larger amplitude + Smaller amplitude)

For oVEMP:

- N10 latency
- P15 latency
- N10 amplitude
- P15 amplitude
- Peak-to-peak latency
- Peak-to-peak amplitude
- RMS (Root Mean Square)
- EMG standard deviation
- Wave reproducibility
- Artifact rejection
- Rosengren formula (2015): Peak-to-peak amplitude / RMS
- Asymmetry ratio = (Larger amplitude – Smaller amplitude) / (Larger amplitude + Smaller amplitude)
