# Supplementary material for: INTerest of electrophysiological and functional EXploration in the evaluation of symptomatic impact of superior semicircular canal DEHIscence syndrome (INTEX-DEHI study): Study protocol for a reliability and validity study
Source: PLoS One. 2025 Sep 18;20(9):e0331763. doi: 10.1371/journal.pone.0331763 (PMC12445554; doi:10.1371/journal.pone.0331763)
Supplement: S2 File — (DOCX) [file pone.0331763.s004.docx]

**INTEX-DEHI**

Version No. 1.0 – 14/04/2022

**Role of Electrophysiological Explorations in the Assessment of Symptom Severity in Superior Semicircular Canal Dehiscence**

**INTEX-DEHI**

**Sponsor Code**

**INTERVENTIONAL RESEARCH PROTOCOL INVOLVING HUMAN PARTICIPANTS**
(Category 2 – minimal risk and burden)

Version No. 1.0 – 14/04/2022

ID-RCB/EudraCT number:

This interventional research was funded by [funding source].

If funded by a Ministry call for projects:
This interventional research received funding from the French Ministry of Health (program name, year of selection, registration number).

**Sponsor:**
Toulouse University Hospital – Department of Research, Development, and Innovation
Hôtel-Dieu St-Jacques
2 rue Viguerie
TSA 80035
31059 TOULOUSE Cedex 9
drci.toulouse@chu-toulouse.fr
Tel: +33 5 61 77 86 03

**Coordinating Investigator:**
Dr. Yohan Gallois
Assistant Professor, Hospital Practitioner, ENT Department
Toulouse University Hospital – Purpan site
Tel: +33 5 61 77 77 04
gallois.y@chu-toulouse.fr

**Methodology and Data Management Center:**
Methodological Research Support Unit (USMR)
Dr. Benoit Lepage
Tel: +33 5 61 14 56 25
lepage.b@chu-toulouse.fr

**Clinical Research Safety Unit:**
Dr. Pascale Olivier-Abbal, PhD
Department of Clinical and Medical Pharmacology
Toulouse University Hospital
Hôtel-Dieu, 2 rue Viguerie, TSA 80039
31059 Toulouse Cedex 9, France
Tel: +33 5 61 14 59 98 / +33 5 61 77 85 56
Email: pascale.olivier@univ-tlse3.fr

This protocol was designed and drafted based on version 4.0 (18/01/2022) of the GIRCI SOHO standard protocol.

**MAIN CONTACTS**

**Principal Investigator**
Dr. Yohan Gallois
University Clinical Chief, Hospital Assistant, ENT Department
CHU Purpan, Toulouse
Tel: +33 5 61 77 77 04
Email: gallois.y@chu-toulouse.fr

**Other Specialties**

**Clinical Research Vigilance Unit**
Dr. Pascale Olivier-Abbal, PhD
Department of Medical and Clinical Pharmacology
CHU de Toulouse
Hôtel-Dieu, 2 rue Viguerie, TSA 80039
31059 Toulouse Cedex 9, France
Tel: +33 5 61 14 59 98 / +33 5 61 77 85 56
Email: pascale.olivier@univ-tlse3.fr

**Sponsor**
Toulouse University Hospital Center
CHU de Toulouse
Hôtel-Dieu, 2 rue Viguerie, TSA 80035
31059 Toulouse Cedex 9, France
Contact: Olivier Lairez
Email: drci.toulouse@chu-toulouse.fr
Tel: +33 5 61 77 86 03
Fax: +33 5 61 77 84 11

**Data Management and Methodology Center**
Methodological Support Unit for Research (USMR)
37, allées Jules Guesde – 31073 Toulouse Cedex
Methodologist: Benoit Lepage
Tel: +33 5 61 14 56 25

**TABLE OF CONTENTS**

**TABLE OF CONTENTS** 4

1. **RESEARCH SUMMARY** 8
   **ABSTRACT** 10
2. **SCIENTIFIC RATIONALE AND GENERAL DESCRIPTION** 12
   2.1. **CURRENT STATE OF KNOWLEDGE** 12
   2.1.1. On the pathology 12
   2.1.2. On reference and study treatments/strategies/procedures 12
   2.2. **RESEARCH HYPOTHESES AND EXPECTED OUTCOMES** 15
   2.3. **RISK/BENEFIT ASSESSMENT** 15
   2.4. **EXPECTED IMPACT** 15
   2.5. **JUSTIFICATION FOR THE LOW LEVEL OF INTERVENTION** 16
3. **RESEARCH OBJECTIVES** 16
   3.1. **PRIMARY OBJECTIVE** 16
   3.2. **SECONDARY OBJECTIVES** 16
4. **OUTCOME MEASURES** 17
   4.1. **CLINICAL MEASURES OF SYMPTOM SEVERITY** 17
   4.1.1. Key symptoms (reference measures) 17
   4.1.1.1. Pulsatile tinnitus 17
   4.1.1.2. Bone-conduction hyperacusis with autophony 17
   4.1.1.3. Vertigo or imbalance induced by loud sounds 18
   4.1.1.4. Vertigo or imbalance induced by pressure changes 18
   4.1.2. Naert questionnaire translated into French 18
   4.1.2.1. Presentation of the questionnaire in English 18
   4.1.3. Quality of life (EQ-5D-5L) 18

4.2. **PARACLINICAL MEASURES** 18
4.2.1. Electrophysiology 18
4.2.1.1. Electrocochleography 19
4.2.1.2. Wideband tympanometry 19
4.2.1.3. VEMP 19
4.2.2. Imaging (CT scan) 19

4.3. **OUTCOME MEASURE CRITERIA: INDICATORS OF VALIDITY AND RELIABILITY** 19
4.3.1. Validity 20
4.3.2. Test-retest reliability (intra-patient) 20
4.3.3. Psychometric evaluation of the Naert questionnaire 20

1. **STUDY DESIGN** 21
   5.1. **JUSTIFICATION OF METHODOLOGICAL CHOICES** 21
   5.2. **STUDY SCHEME** 22
   5.3. **RANDOMIZATION METHODS** 22
2. **ELIGIBILITY CRITERIA** 22
   6.1. **INCLUSION CRITERIA** 22
   6.2. **EXCLUSION CRITERIA** 23
   6.3. **FEASIBILITY AND RECRUITMENT PROCEDURES** 23
3. **RESEARCH TREATMENT(S)/STRATEGY(IES)/PROCEDURE(S)** 23
   7.1. **EXPERIMENTAL TREATMENT/STRATEGY/PROCEDURE** 23
   7.2. **COMPARATOR TREATMENT/STRATEGY/PROCEDURE** 23
   7.3. **PRODUCT HANDLING** 24
   7.4. **BLINDING (if applicable)** 24
4. **ASSOCIATED TREATMENTS AND PROCEDURES** 24
   8.1. **AUTHORIZED ASSOCIATED TREATMENTS/PROCEDURES** 24
   8.1.1. Adjunct medications 24
   8.1.2. Other treatments/procedures 24
   8.2. **PROHIBITED ASSOCIATED TREATMENTS/PROCEDURES** 24
5. **STUDY CONDUCT** 24
   9.1. **STUDY TIMELINE** 24
   9.2. **SUMMARY TABLE OF PARTICIPANT FOLLOW-UP** 24
   9.3. **PRE-INCLUSION/INCLUSION VISIT** 25
   9.3.1. Consent collection 25
   9.3.2. Visit procedure 25
   9.4. **FOLLOW-UP VISITS** 25
   9.5. **END-OF-STUDY VISIT** 26
   9.6. **STOPPING RULES** 26
   9.6.1. For a participant’s withdrawal 26
   9.6.2. For study termination 26
   9.7. **STUDY-RELATED CONSTRAINTS AND POTENTIAL PARTICIPANT COMPENSATION** 27
   9.8. **BIOLOGICAL SAMPLE COLLECTION** 27
   **NOT APPLICABLE – VIGILANCE SECTION FOR CATEGORY 1 STUDIES** 27
6. **MANAGEMENT OF ADVERSE EVENTS / INCIDENTS** 27
7. **STATISTICAL CONSIDERATIONS** 27
   11.1. Sample size calculation 28
   11.2. Statistical methods 28
   11.3. Safety analysis 28
8. **DATA AND SOURCE DOCUMENT MANAGEMENT** 29
   12.1. Data and source documents 29
   12.2. Data collection instructions 29
   12.3. Data handling and flow 29
   12.4. Data confidentiality 29
   12.5. Retention of study documents and data 29
   12.6. Data transfer 30
9. **QUALITY CONTROL AND ASSURANCE** 30
   13.1. Data access 30
   13.2. Quality control 30
   13.3. Audit and inspection 30
10. **ETHICAL AND REGULATORY CONSIDERATIONS** 31
11. **FINAL REPORT** 32
12. **PUBLICATION RULES** 32
    16.1. Scientific communications 32
    16.2. Communication of results to participants 32

**REFERENCES** 33

**LIST OF ABBREVIATIONS**

**AP** – Action Potential
**ANSM** – French National Agency for Medicines and Health Products Safety
**CPP** – Ethics Committee (Comité de Protection des Personnes)
**CRF** – Case Report Form
**DCS** – Superior Semicircular Canal Dehiscence
**EcoG** – Electrocochleography
**EvI** – Adverse Event
**EvIG** – Serious Adverse Event
**EIG** – Serious Adverse Effect
**EIGI** – Unexpected Serious Adverse Effect
**ICH** – International Council for Harmonisation
**MESR** – Ministry of Higher Education and Research
**PEMV** – Vestibular Myogenic Evoked Potentials
**SP** – Summating Potential
**SUSAR** – Suspected Unexpected Serious Adverse Reaction
**WBT** – Wideband Tympanometry

**1. RESEARCH SUMMARY**

| **Section** | **Details** |
| --- | --- |
| **SPONSOR** | CHU de Toulouse, Hôtel-Dieu, 2 rue Viguerie, 31052 Toulouse Cedex 9Phone: +33 5 61 77 86 03Fax: +33 5 61 77 84 11 |
| **PRINCIPAL INVESTIGATOR / COORDINATOR** | Dr. Yohan GalloisUniversity Clinical Chief, Hospital Assistant, ENT DepartmentCHU Purpan, ToulousePhone: +33 5 61 77 77 04Email: [gallois.y@chu-toulouse.fr](mailto:gallois.y@chu-toulouse.fr) |
| **TITLE** | INTEX-DEHI: Relevance of Electrophysiological Investigations in Assessing Symptomatic Impact of Superior Semicircular Canal Dehiscence (SSCD) |
| **JUSTIFICATION / BACKGROUND** | Superior semicircular canal dehiscence (SSCD) is a major treatable cause of tinnitus and hyperacusis. Complete SSCD syndrome (Minor’s syndrome) presents four cardinal auditory and vestibular symptoms: Pulsatile tinnitusBone-conduction hyperacusis with autophonyVertigo or imbalance induced by loud soundsVertigo or imbalance induced by pressure changes Symptom presentation is heterogeneous in type and severity. Radiological prevalence of SSCD ranges from 2–9%, suggesting most cases are minimally or asymptomatic. SSCD acts as a “third window” between the inner and middle ear, altering labyrinthine fluid biomechanics and reducing vestibular impedance. Diagnosis relies on high-resolution CT of the temporal bone with specific reconstructions. Vestibular myogenic evoked potentials (VEMPs) can support diagnosis if imaging is inconclusive. Currently, no objective markers exist to quantify symptom severity, complicating therapeutic decisions. Surgical treatment achieves 70–90% success, but failures (10–30%) and auditory complications (up to 25%) are significant. Objective symptom severity markers could guide therapy. |
|  | Wideband tympanometry (WBT) and electrocochleography (EcoG) are non-invasive electrophysiological tests recently evaluated in SSCD patients. WBT measures middle and inner ear impedance via energy absorbance peak at 1 kHz in SSCD. EcoG measures inner ear impedance via the summating potential/action potential (SP/AP) ratio, abnormally elevated in cochlear pressure imbalance. Post-surgical normalization of SP/AP correlates with symptom improvement. EcoG is therefore promising as an objective marker of symptom severity. |
| **PRIMARY OBJECTIVE** | Evaluate EcoG as an objective marker of SSCD symptom severity by studying the association between SP/AP ratio elevation and the severity of each cardinal symptom (pulsatile tinnitus, autophony, sound-induced balance disturbances, pressure-induced balance disturbances). |
| **SECONDARY OBJECTIVES** | Compare associations of SP/AP ratio with symptom severity to associations of VEMP abnormalities, WBT absorbance peaks, and CT-based SSCD characteristics.Assess relationships between anatomical/radiological SSCD features and SP/AP, VEMP, and WBT parameters.Evaluate validity of SP/AP, VEMP, and WBT in relation to quality of life (EQ-5D-5L).Evaluate combined electrophysiological indicators (EcoG ± WBT ± VEMP) for symptom assessment.Assess validity of each individual indicator against Naert symptom severity score (French translation).Identify patient profiles based on vestibular/auditory symptom predominance and objective electrophysiological results.Evaluate reliability of electrophysiological indicators (EcoG, VEMP, WBT) over repeated measures (3 visits, 6 measures) by the same examiner.Validate the French version of Naert’s symptom severity questionnaire. |
| **OUTCOME MEASURES** | **Clinical:** Visual analog scales for each cardinal symptom, Naert questionnaire (French), EQ-5D-5L.**Electrophysiological:** EcoG (SP/AP ratio), VEMP (threshold reduction, abnormally large potentials), WBT (absorbance peak at 1 kHz).**Radiological:** SSCD size, surface, and localization on CT. **Primary endpoint:** Correlation coefficients between SP/AP ratio and symptom severity (visual analog scales) for affected vs. healthy side.**Secondary endpoints:** Correlations between VEMP/WBT/CT abnormalities and symptoms, correlations with quality of life, patient profile identification via PCA and clustering, and reliability/reproducibility assessments using ICC and Bland-Altman methods. |
| **STUDY DESIGN** | Prospective, national, multicenter cohort study of patients with unilateral SSCD confirmed on CT. Descriptive analysis of clinical and paraclinical features and correlations. **Reliability assessment:** 3 visits (<1 month apart), 2 repeated measures per visit, standardized procedures, single trained operator per center. Test-retest reliability of French Naert questionnaire over 3 visits (<3 weeks apart). |
| **INCLUSION CRITERIA** | Unilateral SSCD confirmed on high-resolution CT (0.5 mm slices, specific reconstructions)If present, stable SSCD-related symptoms with no treatment planned within 3 monthsAdults (≥18 years), French-speakingAffiliated with a social security schemeSigned informed consent by participant and investigator |
| **EXCLUSION CRITERIA** | Bilateral SSCD (to avoid symptom attribution ambiguity). Doubtful CT diagnosisAssociated otological/otoneurological conditions affecting symptoms (chronic otitis, otosclerosis, Meniere’s disease, vestibular migraine)Legal protection (guardianship, curatorship) Pregnancy |
| **INTERVENTIONS / PROCEDURES** | Study links objective SSCD anomalies (electrophysiological or radiological) with symptom severity. Non-invasive, routine tests: **EcoG:** Cochlear and nerve responses via tympanic electrode to controlled auditory stimulation**WBT:** Absorbed acoustic energy in middle ear via wideband probe**VEMP:** Muscle responses to high-intensity auditory stimulation via neck/lower eyelid electrodes |
| **SAMPLE SIZE** | 100 patients with unilateral radiologically confirmed SSCD |
| **STUDY DURATION** | Inclusion period: 18 monthsParticipant follow-up: 1 monthTotal study duration: 19 months |
| **STATISTICAL ANALYSIS** | Reliability: Bland-Altman plots (bias and 95% limits of agreement), ICC. Convergent/divergent validity: average of repeated measures over 3 visits. Multivariate descriptive analysis (PCA, clustering). Supervised modeling (partial least squares regression) for symptom prediction based on electrophysiological ± anatomical indicators. |
| **EXPECTED OUTCOMES** | Better understanding of SSCD pathophysiology, symptom expression, and vestibular/auditory predominance via electrophysiological explorationFrench validation of Naert questionnaire for patient-perceived care quality assessmentIdentification of objective symptom severity markers for future therapeutic studiesFirst national multicenter study in this field, increasing visibility of French otology teams in a research area dominated by North America |

Abstract

This research has been registered in <http://www.clinicaltrials.gov/> the *date* under the n° *numéro.*

**Interests of electro-physiological findings in the assessment of symptoms severity in superior semi-circular canal dehiscence syndrom (INTEX-DEHI)**

**Role of electro-physiological findings in the assessment of symptoms severity in Minor syndrom**

University Hospital Toulouse is the sponsor of this research.

This research will be conducted with the support of *nom de la firme pharmaceutique / source of grants (PHRC,…).*

- **Brief summary :** this research aims at assessing the validity of three different electro-physiological tests (Vestibular Evoked Myogenic Potentials, WideBand Tympanometry, Electrocochleography) used in the investigation of the symptoms severity in the case of superior semi-circular canal dehiscence syndrom*.*
- **Detailed description :** Superior semi-circular canal dehiscence (SCD) syndrom can associate several characteristic auditory and vestibular symptoms**.** Recent systematic reviews identified pulsatile tinnitus, autophony, sound-induced and pressure-induced vertigo as most specific signs of SCD. These signs are rarely all present and the symptomatology remains highly variable from a patient to another. A surgical treatment may be proposed when the symptoms are significantly incapacitating. Unfortunately to date, there is no objective marker of this severity and all surgical decisions rely on the importance of patients’ complaints. High Resolution Computed Tomography (HRCT) of the temporal bone can confirm the diagnosis. Electro-physiological investigations such as Vestibular Evoked Myogenic potentials (VEMPs) have also been extensively described in the diagnosis of SCD but they have been reported as independant from the symptoms severity. Wideband tympanometry (WBT) and Electrocochleography (EcoG) have only been studied in few reports but the latter appears as a promising tool in the assessment of the symptoms because it explores inner ear biomechanics, directly involved in the pathophysiology of this condition.

These three electrophysiological tests are non invasive, commonly used in a routine neurotology practise, and provide objective findings that may be studied in relation to the subjective symptoms reported by the patients, on a visual analogue scale and on a questionnaire translated in French from Naert et al. (2021).

Three visits will be planned within one month +/-7 days period. Each visit will take place 15 days after the previous one and will include questionnaires on SCD symptoms severity (visual analogue scale and questionaire from Naert), the three electro-physiological tests (VEMPs, WBT, EcoG) repeated twice.

- **Primary outcome**: the validity of EcoG will be assessed by the correlation between the alteration of the summating potential/action potential ratio and each symptom severity assessed by a visual analogue scale (mean correlation for the three visits)
- **Secondary outcomes:**

-the validity of VEMPs, WBT and HRCT will be assessed by the association between their alteration (thresholds and amplitude for VEMPs, 1 kHz absorbance peaks for WBT, size and surface of SCD on HRCT) and each symptom severity

-the validity of EcoG, VEMPs, WBT and HRCT will be also assessed by the association between their alteration and the quality of life of patients, evaluated using EuroQoL-EQ5D-5L

-the reliability of EcoG, WBT and VEMPs using intra-class correlation coefficients and Bland and Altmann analyses

-the validation of French version of Naert questionnaire will be made using a standardized procedure with analysis of internal consistency using Crohnbach coefficient, of inter and total-items correlation, factorial analysis, test-retest reliability using intra-class correlation coefficient and Bland and Altman method

- **Study design** : Multicentre, National, prospective cohort study of 100 patients with confirmed unilateral SCD to describe clincal, radiological and electro-physiological characteristics and their correlation

Evaluation of the reliability of the electro-physiological tests (VEMPs, WBT, EcoG) using repeated measures with two measures performed by visit, for three visits. All the measures will be made on a standardized material, using standardized procedure, by one trained examinator

Assessment of test-retest reliability of translated version of Naert’s questionnaire based on repeated measures across the three visits

- **Eligibility criteria:**
  - inclusion criteria:
    - *confirmed unilateral SCD (using specific HRCT settings and reconstructions) with or without SCD symptoms*
    - if present, SCD symptoms should be stable
    - *adult (>18 years old) patients, understanding and reading french*
    - *affiliated to social security.*
  - exclusion criteria:
    - *bilateral confirmed SCD*
    - *doubtful SCD*
    - *other otologic or neurotologic condition that might mimick SCD symptomatology : middle ear conditions, Meniere disease, vestibular migraine*
- **Arm number or label and arm type** : *NA*
- **Interventions :**
- **Number of subjects :** *100 subjects will be included.*
- **Statistical analysis :** The reliability of repeated measures (electrophysiological, clinical symptoms, Naert's questionnaire) will be assessed using the graphical method of Bland and Altman (and estimates of systematic bias and 95% concordance limits), as well as by estimating intra-class correlation coefficients.

For the study of convergent and divergent validity, the average of the measurements repeated during the 3 follow-up visits will be used. For convergent (respectively divergent) validation, the analysis of correlations between the electrophysiological indicators measured on the SCD side (respectively on the healthy side) and the symptoms (presence, intensity, Naert scale) will be carried out*.*

- **Conditions :** Superior semi-circular canal dehiscence. Minor syndrom. Pulsatile tinnitus. Autophony.
- **Key-words :** Superior semi-circular canal dehiscence. Minor syndrom. Pulsatile tinnitus. Autophony. Tullio phenomenon

**2. SCIENTIFIC JUSTIFICATION AND GENERAL DESCRIPTION**
“I hear my eyes moving and my heart beating in my ear.” “I see the floor shaking when I walk.” Lloyd Minor, an otologist and otoneurosurgeon, now Dean of the Stanford University School of Medicine in the United States, often recounts the months or even years spent trying to match a lesion with an unusual combination of symptoms. Superior semicircular canal dehiscence (SCD) syndrome was finally described for the first time in 1998 by Minor and his team. Nearly 25 years later, the pathology remains relatively unknown, both to the general public and within routine ENT practice. Patients’ journeys are often marked by long diagnostic odysseys, which can last for years (Eberhard et al., 2021).

**2.1. CURRENT STATE OF KNOWLEDGE**

**2.1.1. ABOUT THE PATHOLOGY**
This diagnostic delay is particularly regrettable since superior semicircular canal dehiscence (SCD) represents one of the main treatable causes of vertigo, tinnitus, and hyperacusis (Houdart, 2021). In its most classic form, SCD syndrome combines four cardinal signs, present both in the auditory and vestibular domains (Eberhard et al., 2021):

- Pulsatile tinnitus
- Bone-conduction hyperacusis with autophony (abnormally intense perception of body sounds and the patient’s own voice) and supra-normal bone conduction measured in pure-tone audiometry (with slightly reduced air conduction, incorrectly suggesting conductive hearing loss)
- Vertigo or imbalance induced by loud sounds
- Vertigo or imbalance induced by pressure changes

However, the simultaneous presence of all these characteristic signs is rare. Patient history often reports multiple less-specific complaints such as instability, a feeling of ear pressure, or whistle-type tinnitus (Naert et al., 2021). Beyond the heterogeneity of symptom types, there is significant variability in their impact, with some patients showing radiological SCD without symptoms, while others are severely impaired, representing a continuum in symptom severity. Naert et al. recently developed and validated a questionnaire to record and grade most symptoms reported by these patients, a future tool to assess patient-perceived quality of care in this pathology.

The overall lack of awareness limits understanding of its true prevalence, but radiological prevalence is estimated between 2% and 9% among patients undergoing temporal bone CT (Williamson et al., 2003; Masaki, 2003; Stimmer et al., 2012; Berning et al., 2019). This leads to diagnostic delays and impaired quality of life for many patients (Remenschneider et al., 2015), particularly regrettable since surgical treatment yields favorable outcomes in 70–90% of cases depending on series (Nieto et al., 2021; Rodgers et al., 2016; Ziylan et al., 2016).

The classically accepted pathophysiological hypothesis to explain symptoms is the presence of a “third window” in the inner ear, created by the bony defect of the superior semicircular canal, in addition to the physiological oval and round windows (see Figure 1).

**Figure 1:** Left superior semicircular canal dehiscence (red arrow), showing a bony defect on a coronal temporal bone CT slice, confirmed after reconstruction in its plane.

This third window is thought to cause localized impedance changes in the vestibular compartment of the inner ear, leading to auditory and vestibular symptoms. Reduced impedance in the vestibular compartment would dissipate acoustic energy (responsible for conductive hearing loss), cause hyperexcitability of the basilar membrane via bone conduction (responsible for autophony), and hyperexcitability of the superior canal and vestibular organs (responsible for balance disorders, regardless of form). However, this hypothesis does not account for the significant proportion of asymptomatic subjects with radiologically confirmed SCD, nor does it explain the predominance of either auditory or vestibular symptoms.

Radiological (temporal bone CT) and electrophysiological investigations play an important role in SCD diagnosis (see below, 2.1.2 Procedures under study). CT can visualize SCD when performed with 0.5 mm fine slices and specific reconstructions in the plane of the superior semicircular canal (Pöschl plane) and the perpendicular plane (Stenvers plane), possibly complemented by pixel density variations along the canal roof (Schwartz et al., 2021). Vestibular Evoked Myogenic Potentials (VEMPs) are the main electrophysiological tool for routine SCD diagnosis and classically show reduced thresholds and increased response amplitudes to auditory stimulation. Their sensitivity and specificity remain limited, around 70% (Noij et al., 2020). Other tests potentially useful for SCD evaluation (electrocochleography and wideband tympanometry) have been less studied. Wideband tympanometry (WBT) can reveal an absorbance peak around 1 kHz (Nakajima et al., 2012; Merchant et al., 2015), and electrocochleography (EcoG) an abnormally high summating potential to action potential (SP/AP) ratio.

Ultimately, SCD diagnosis relies on a combination of clinical findings and para-clinical evidence. A recent literature review published by the Bárány Society in the context of vestibular disorder classification (Ward et al., 2021) states that SCD syndrome can be diagnosed if the following criteria are met:

- At least one clinical sign of a third window:
  - Pulsatile tinnitus
  - Bone-conduction hyperacusis with autophony
  - Vertigo or imbalance induced by loud sounds
  - Vertigo or imbalance induced by pressure changes
- At least one para-clinical test indicative of a third window:
  - Nystagmus triggered by intracranial or middle ear pressure changes (e.g., Valsalva maneuvers)
  - Supra-normal bone conduction at low frequencies
  - Abnormally large VEMPs or abnormally low thresholds
  - Visualization of SCD on high-resolution temporal bone CT
- No other suspected auditory or vestibular disorder

Once diagnosed, patient management depends on symptom severity, as reported by patients, with no objective assessment tool currently available:

- Severely affected patients may be offered surgery to repair or cover the superior semicircular canal, though 10–30% may experience insufficient symptom improvement.
- Less affected patients or those wishing to avoid surgery receive education on the pathology and instructions to avoid symptom triggers (e.g., loud sounds, sports).

The lack of an objective marker may explain why 10–30% of patients remain insufficiently improved after surgical treatment (Nieto et al., 2021; Rodgers et al., 2016; Ziylan et al., 2016), which is not without risks, with up to 25% iatrogenic hearing loss reported in some series (Ward et al., 2012).

Clinicians therefore still lack a tool to objectively determine symptom severity (primary objective of this project). Once identified, its relevance for guiding patient management could be evaluated in a subsequent study.

**2.1.2. ABOUT TREATMENTS / REFERENCE STRATEGIES / PROCEDURES UNDER STUDY**
An objective marker of symptom severity could logically be sought among results from para-clinical diagnostic investigations. However, to date, available tests—from temporal bone CT to electrophysiological tests—have not explained symptom heterogeneity.

The relationship between the radiological size of the dehiscence and symptom severity was initially debated (Chien et al., 2012; Pfammater et al., 2010), and recent literature reviews even discount this correlation (Eberhard et al., 2021; Ward et al., 2021). Electrophysiological tests (VEMPs, WBT, EcoG) are routinely used in otology or otoneurology consultations to explore conditions such as Ménière’s disease (WBT and EcoG), perilymphatic fistulas (WBT), or suspected auditory neuropathies (EcoG). These non-invasive tests each take less than 15 minutes. Theoretically, tests most capable of measuring inner ear impedance changes should best assess the objective impact of SCD.

**2.1.2.1 Vestibular Evoked Myogenic Potentials (VEMPs)**
VEMPs assess the function of the two otolith organs in the vestibule: the utricle and saccule. Utricular function is assessed by ocular VEMPs via the utriculo-ocular pathway, using short high-intensity auditory stimuli (500 Hz and/or 2000 Hz tones at 90 dB SPL) delivered via headphones to the test ear and recorded with a surface electrode under the contralateral lower eyelid. Saccular function is assessed by the cervico-collic reflex, with inhibitory muscular responses recorded from the ipsilateral sternocleidomastoid.

In unilateral SCD, VEMP thresholds (ocular and cervical) are lowered and amplitudes increased compared to the unaffected ear, reflecting vestibular hyper-reflexivity. VEMP sensitivity and specificity in SCD diagnosis exceed 70% (Hunter et al., 2017; Noij et al., 2020). Some authors have proposed combining VEMPs with low-frequency conductive hearing loss as a “third window indicator” with 92% sensitivity and 100% specificity (Noij et al., 2018).

VEMP abnormalities can aid SCD diagnosis, especially in ambiguous CT cases, but do not correlate with symptom type or severity (Bae JS et al., 2013; Noij et al., 2018). No standardized thresholds exist, and post-surgical VEMP interpretation can be challenging due to utricular or saccular deficits.

**Figure 2:** Example of cervical VEMPs with abnormal lowered thresholds on the left side.

**2.1.2.2 Wideband Tympanometry (WBT)**
WBT non-invasively measures middle and inner ear mechano-acoustic impedance using a probe delivering a 226–8000 Hz stimulus and measuring reflected energy to calculate absorbance. SCD reduces inner ear impedance, detectable as an absorbance peak at 1 kHz, provided middle ear function is normal. Nakajima et al. (2012) and Merchant et al. (2015) reported WBT sensitivity of 80–93% for SCD diagnosis. No study has yet correlated the 1 kHz absorbance peak with symptom severity.

**2.1.2.3 Electrocochleography (EcoG)**
EcoG non-invasively records inner ear responses using a tympanic electrode. Key measures include the summating potential (SP) from hair cells and action potential (AP) from auditory neurons, with the SP/AP ratio often analyzed. SP/AP may increase in hair cell polarization anomalies, as seen in Ménière’s disease. Inner ear biomechanical changes in SCD may also elevate SP/AP.

Recent studies (Park et al., 2015; Wenzel et al., 2015; Adams et al., 2011) indicate EcoG as a promising objective marker of SCD symptom impact, with SP/AP decreasing post-surgical canal occlusion in symptomatic patients, correlating with clinical improvement. Normal SP/AP values are consensus-defined (<0.25 at >70 dB SPL, <0.35 at 40–70 dB SPL).

**2.2. RESEARCH HYPOTHESES AND EXPECTED RESULTS**
Based on current pathophysiological consensus and literature:

- Inner ear impedance changes likely explain symptom severity in SCD.
- High-resolution temporal bone CT and VEMPs are commonly used to confirm diagnosis but do not correlate with symptom severity.
- Among electrophysiological tests, WBT evaluates middle and inner ear impedance; EcoG assesses inner ear impedance changes.

We hypothesize in adult patients with unilateral radiologically confirmed SCD:

- Symptom severity (cardinal and questionnaire-measured) will:
  - Be weakly or not associated with CT-measured dehiscence
  - Be weakly or not associated with VEMP alterations
  - Be weakly associated with WBT absorbance peak
  - Be strongly associated with elevated SP/AP ratio in EcoG

Thus, we propose:

- SP/AP elevation is a valid objective marker of SCD symptom impact
- SP/AP elevation correlates more strongly with symptom severity than anatomical CT findings, VEMP alterations, or 1 kHz WBT absorbance peak

If confirmed, this marker could guide therapeutic decisions, complementing current symptom-severity-based management.

**2.3. BENEFIT / RISK RATIO**
Study risks are low. Electrophysiological tests are routine, non-invasive, and may cause minor discomfort from probe insertion or electrode placement. No additional adverse effects are expected. Participants will not gain direct personal benefit beyond more comprehensive exploration and understanding of their symptoms.

**2.4. EXPECTED OUTCOMES**
This study has several novel aspects:

- Inclusion of rarely studied pauci- or asymptomatic patients
- First study combining three electrophysiological tests typically performed separately
- Potential new insights on test reliability through repeated measures

Patient benefits include:

- Identification of objective markers for symptom impact, enhancing pathophysiological understanding
- Potential future use of these markers to guide therapy
- French validation of Naert et al.’s (2021) questionnaire as a patient-perceived quality-of-care tool

Strategically, this will be the first national multicenter study on SCD, increasing visibility for French otology teams in a field largely dominated by North American research.

**2.5. JUSTIFICATION FOR LOW-INTERVENTION LEVEL**
This study primarily involves non-invasive electrophysiological tests and questionnaires in SCD patients, each taking under 15 minutes. Combining these procedures with quality-of-life questionnaires justifies a category 2 human research study.

**3. RESEARCH OBJECTIVES**

**3.1. PRIMARY OBJECTIVE**
The primary objective of this study is to evaluate the validity of electrocochleography as an objective marker of symptom impact in SCD by examining the association between the elevation of the SP/AP ratio on the side of the SCD and the severity of each cardinal symptom (pulsatile tinnitus, autophony, sound-induced vertigo, pressure-induced vertigo).

**3.2. SECONDARY OBJECTIVES**

- To compare the observed association between the elevation of the SP/AP ratio in electrocochleography and symptom severity with the respective associations observed between PEMV alterations and symptom severity, wideband tympanometry and symptom severity, temporal bone CT and symptom severity (evaluated for each cardinal symptom).
- To evaluate the associations between the anatomical-radiological characteristics of SCD (size, location) on temporal bone CT and:
  - the elevation of the SP/AP ratio in electrocochleography,
  - PEMV alterations (lowered PEMV threshold, abnormally large PEMV),
  - and changes in the absorbance peak in wideband tympanometry.
- To assess the validity of SP/AP ratio elevation in electrocochleography, PEMV threshold lowering, and absorbance peak changes in wideband tympanometry, on the SCD side, in relation to quality of life as measured by a generic questionnaire (Euro-QoL 5D).
- To evaluate the validity of combined electrophysiological indicators (EcoG +/- WBT +/- PEMV) in assessing the symptom impact of SCD.
- To evaluate the validity of each indicator (SP/AP ratio in EcoG, PEMV threshold lowering, absorbance peak changes in WBT, and anatomical-radiological characteristics of SCD) by studying their association with symptom severity scores measured using the French translation of the Naert et al. questionnaire.
- To identify multiple patient profiles according to the coexistence or predominance of vestibular and auditory symptoms, and the results of objective electrophysiological tests (PEMV, WBT, EcoG).
- To evaluate the reliability of these electrophysiological indicators by repeating their measurement by the same examiner:
  - over 3 successive visits (in a clinically stable diagnostic and follow-up context, with no therapeutic intervention between visits) in an outpatient setting within a 1-month interval, allowing assessment of inter-visit reliability,
  - with 2 measurements performed at each of the 3 visits by the same examiner, allowing assessment of intra-visit reliability.
    More broadly, these 6 measurements will be used to assess intra-patient reliability.
- To validate the Naert et al. (2021) symptom severity questionnaire in French.

**4. OUTCOME MEASURES**

This study aims to evaluate the validity of objective para-clinical assessments (electrophysiological and radiological) in measuring the symptom impact of SCD. This objective is based on the correlation of clinical indicators of symptom severity with para-clinical indicators in a construct validation approach according to the hypotheses described in section 2.2, with:

- convergent validity, by verifying positive correlations between symptom impact and electrophysiological or radiological measurements on the pathological side,
- discriminant validity (or divergent validity), by verifying the absence of correlation between symptom impact and electrophysiological or radiological measurements on the healthy side.

All clinical and para-clinical measurements are detailed below, before presenting the validity and reliability outcome criteria for:

- the validity of EcoG and other objective para-clinical assessments (temporal bone CT, PEMV, WBT),
- the reliability of electrophysiological assessments (EcoG, PEMV, WBT),
- the psychometric properties of the Naert questionnaire translated into French.

**4.1. CLINICAL MEASURES OF SYMPTOM SEVERITY**

**4.1.1. CARDINAL SYMPTOMS (REFERENCE MEASURES)**
Cardinal symptoms, the Naert questionnaire, and quality of life will be assessed at each visit.

**4.1.1.1. PULSATILE TINNITUS**
The presence and severity of unilateral pulsatile tinnitus on the SCD side will be assessed using a visual analog scale (VAS) ranging from 0 (no pulsatile tinnitus) to 100 (extremely disabling pulsatile tinnitus) at each of the three planned visits.

**4.1.1.2. BONE-CONDUCTED HYPERACUSIS WITH AUTOPHONY**
The presence and severity of autophony on the SCD side will be assessed using a VAS ranging from 0 (no autophony) to 100 (extremely disabling autophony) at each of the three planned visits.

**4.1.1.3. SOUND-INDUCED VERTIGO OR INSTABILITY**
The presence and severity of sound-induced balance disorders (vertigo and/or instability) will be assessed using a VAS ranging from 0 (no sound-induced balance disorder) to 100 (extremely disabling sound-induced balance disorder) at each of the three planned visits.

**4.1.1.4. PRESSURE-INDUCED VERTIGO OR INSTABILITY**
The presence and severity of pressure-induced balance disorders (vertigo and/or instability) will be assessed using a VAS ranging from 0 (no pressure-induced balance disorder) to 100 (extremely disabling pressure-induced balance disorder) at each of the three planned visits.

**4.1.2. NAERT QUESTIONNAIRE TRANSLATED INTO FRENCH**

**4.1.2.1. QUESTIONNAIRE DESCRIPTION IN ENGLISH**
The scale, in both English and translated French versions, consists of 31 items measuring the severity of 22 symptoms reported in SCD, identified from a systematic literature review (Naert et al., 2018). Responses are recorded on a 5-point Likert scale from 0 (no problem) to 4 (as severe as possible).

The original English questionnaire is reproduced below.

The Cronbach alpha coefficient was estimated at 0.969 in a sample of 29 SCD patients and 58 controls (Naert et al., 2021). The French translation method is presented in section 5, Study Design.

**4.1.3. QUALITY OF LIFE (EQ5D-5L)**
The EQ5D-5L is a generic quality-of-life questionnaire consisting of 5 questions on:

- Mobility,
- Self-care,
- Usual activities,
- Pain/discomfort,
- Anxiety/depression.

Responses use a 5-level Likert scale. A self-rated health measure is also taken using a visual analog scale (0–100): “How good or bad is your health today?”

**4.2. PARACLINICAL MEASURES**

**4.2.1. ELECTROPHYSIOLOGY**
Electrophysiological tests will be performed twice during each of the three visits, for a total of 6 measurements.

**4.2.1.1. ELECTROCOCHLEOGRAPHY**
Two EcoG measurements are performed at each of the three planned visits, recording summation potential (SP) and action potential (AP) at each measurement (see Figure 3). The SP/AP ratio is calculated for each of the 6 measurements, as well as an average SP/AP ratio per visit and an overall average across all 6 measurements.

*Figure 3: Electrode placement and typical EcoG recording (from Interacoustics Academy)*

All recordings are performed by the same examiner within one center using the EcoG module of the Eclipse® modular system by Interacoustics®.

**4.2.1.2. WIDEBAND TYMPANOMETRY (WBT)**
Two WBT recordings are performed at each of the three visits in response to wideband auditory stimulation (226–8000 Hz). They allow determination of the absorbance peak for each of the 6 measurements (see Figure 4), with the frequency of the peak recorded. The proportion of absorbance peaks at 1 kHz (associated with SCD in the literature) across the 6 measurements is also recorded.

*Figure 4: Procedure and typical 1 kHz absorbance peak in SCD (from Eberhard, 2021)*

All recordings are performed by the same examiner within one center using the Titan® tympanometry system (Interacoustics®).

**4.2.1.3. PEMV**
Two ocular and cervical PEMV recordings are performed at each of the three visits, collecting the PEMV threshold (dB SPL) and wave amplitude characterization relative to the contralateral healthy side (absent, normal, or abnormally large). PEMV thresholds and the proportion of abnormally large PEMV across the 6 measurements are recorded.

All recordings are performed by the same examiner within one center using the EcoG module of the Eclipse® system (Interacoustics®).

In each center, the examiner responsible for electrophysiological assessments will have received standardized training by Mr. Fabrice Giraudet (Associate Professor, University Clermont-Auvergne, and consultant to Interacoustics®).

**4.2.2. CT SCAN**
Formal evidence of SCD is a main inclusion criterion. Temporal bone CT is performed before inclusion using high-resolution scans (≤0.5 mm slices), with reconstructions in the plane of the superior semicircular canal and its perpendicular plane (see Figure 5).

*Figure 5: Reconstructions of the superior semicircular canal and perpendicular plane confirming SCD*

SCD is characterized by loss of bone hyperdensity over at least two consecutive slices (Lookabaugh et al., 2015). Its location (anterior limb, arch, posterior limb), largest dimension in mm, and surface area in mm² are recorded.

**4.3. OUTCOME MEASURES: VALIDITY AND RELIABILITY INDICATORS**

**4.3.1. VALIDITY**
Main indicators for evaluating convergent and discriminant validity of electrophysiological or radiological parameters relative to clinical parameters will be Pearson correlation coefficients (or standardized regression coefficients). According to the pathophysiological hypotheses in section 2.2, we expect:

- Significant positive correlation between the SP/AP ratio in EcoG and severity of each cardinal symptom measured on the corresponding VAS.
- Higher correlation between the pathological-side SP/AP ratio and each cardinal symptom than between SCD anatomical size and surface, proportion of 1 kHz absorbance peaks in WBT, or PEMV alteration and symptom severity measured on the corresponding VAS.
- No correlation between cardinal symptom severity and electrophysiological (or radiological) indicators on the healthy side.
- Moderate correlation between anatomical SCD measurements on CT and PEMV alteration or WBT 1 kHz absorbance peak presence.

Correlation coefficients will also be calculated between combined electrophysiological criteria (EcoG +/- WBT +/- PEMV) and each cardinal symptom severity, between electrophysiological criteria and generic quality of life (EQ-5D-5L), and between electrophysiological criteria and symptom severity on the French Naert questionnaire.

Patient profiles with unilateral SCD will be determined using principal component analysis, supplemented by supervised modeling.

**4.3.2. TEST-RETEST RELIABILITY (INTRA-PATIENT)**
To evaluate electrophysiological reliability, measurements will be repeated 6 times (2 per visit, across 3 visits within one month).
A trained operator (same in each center) will perform all electrophysiological tests using standardized equipment identical across visits.

Indicators for intra-patient (6 repeated measures), intra-visit (2 repeated measures), or inter-visit (3 repeated measures) reliability will include:

- Bland-Altman plots describing the differences between repeated measures vs. their mean. This visually evaluates absolute differences, systematic bias, bias variation, and variance of differences. Mean systematic bias (average difference) with 95% CI and 95% limits of agreement will be estimated (Bland & Altman, 1999).
- Intraclass correlation coefficients (ICC) for intra-patient, intra-visit, and inter-visit reliability.

**4.3.3. PSYCHOMETRIC EVALUATION OF THE NAERT QUESTIONNAIRE**
Using the French-translated scale, the following properties will be assessed:

- Internal consistency (Cronbach’s alpha coefficient),
- Inter-item and total-item correlation coefficients,
- Factor analysis to evaluate unidimensionality,
- Test-retest reliability across 3 repeated measures (one per visit, in clinically stable conditions) using Bland-Altman method and ICC calculation.

**5. STUDY DESIGN**

**5.1. JUSTIFICATION OF METHODOLOGICAL CHOICES**
In this study, the objective is to evaluate the validity and reliability of various electrophysiological parameters to better and objectively identify the severity of symptom impact in SCD.

The reliability of a measurement sets the upper limit of the maximum validity it can achieve (Streiner, 2015). The methodological choices made to address the objectives are as follows:

- Selection of patients with unilateral SCD formally diagnosed by temporal bone CT: patients with bilateral SCD will not be included to avoid uncertainty in attributing symptoms to one, the other, or both SCDs.
- No requirement for symptom severity, to obtain a population sufficiently heterogeneous in terms of symptom impact and representative of patients followed in ENT hospital services.
- Stable SCD in a diagnostic and follow-up context, with no therapeutic intervention planned within 3 months of the first visit. This allows evaluation of the test-retest reliability of different electrophysiological parameters, with measurements repeated over 3 successive visits (less than one month apart) and 2 repeated measurements at each visit. These repeated measures will allow assessment of intra-patient reliability (6 repeated measurements), intra-visit reliability (2 repeated measurements per visit), and inter-visit reliability (3 repeated visits).

The evaluation of the validity of electrophysiological measurements will primarily rely on a convergent and divergent validation approach by estimating the associations between each electrophysiological parameter and patient-reported symptoms (cardinal symptoms, Naert questionnaire, and quality of life). The main hypothesis (based on the theoretical model from the literature) is that the SP/AP ratio measured in electrocochleography will be the parameter most strongly correlated with the cardinal symptoms, compared to other electrophysiological parameters. More generally, all correlations obtained in this study between clinical and paraclinical measures will provide a better understanding of the construct validity of the various electrophysiological parameters.

The Naert questionnaire is not yet validated in French. A translation of the scale is underway, and the psychometric properties of this new translated version will be evaluated during this study. Repeated administration of the questionnaire over the 3 follow-up visits will allow assessment of test-retest reliability.

The Naert questionnaire translation follows the recommendations described by Hall et al. (2017). The corresponding author (V. Van Rompaey) has authorized the use of this questionnaire in our study. The translation from English to French was performed by two bilingual translators with French as their native language (one professional translator, one healthcare professional), and an intermediate French version of the questionnaire was harmonized. This intermediate version is currently undergoing back-translation by a third bilingual translator with French as their native language, and corrections will be made if discrepancies are significant.

A review committee including two SCD expert clinicians (Dr E. Ionescu, Prof. M. Marx), the three translators, and an expert patient with SCD will be convened to validate the version to be tested in the field. This version will be submitted to a group of 8–10 SCD patients, representative of the symptomatic variability observed in practice, to assess comprehension and ease of completion and to finalize a validated questionnaire before study initiation. The formal validation study of the final translated version will then be conducted on the 100 patients included in this project, with evaluation of its psychometric properties.

**5.2. STUDY SCHEMA**
Cohort study of patients with unilateral SCD diagnosed by CT,

- prospective,
- national, multicenter,
- descriptive of clinical and paraclinical characteristics of the pathology and their correlations.

Evaluation of the reliability of electrophysiological parameters relies on repeated measurements:

- across 3 visits (less than one month apart),
- two repeated measurements per visit, following standardized procedures on standardized equipment, performed by a single trained operator at each center.

Test-retest reliability of the Naert questionnaire (French translated version) is based on repeated measurements across 3 successive visits (less than one month apart).

**5.3. RANDOMIZATION METHODS**
No randomization procedure is planned in this study.

**6. ELIGIBILITY CRITERIA**

**6.1. INCLUSION CRITERIA**

- Unilateral SCD formally diagnosed by high-resolution CT (≤0.5 mm slices, reconstructions in the plane of the superior semicircular canal and perpendicular plane) of the temporal bone, with no requirement for symptom severity.
- If present, SCD-related symptoms must be stable in a diagnostic and follow-up context, with no therapeutic intervention planned within 3 months of the first visit.
- Adult patient (≥18 years), able to read and understand French.
- Affiliated with or beneficiary of a social security scheme.
- Free, informed, written consent signed by the participant and investigator (at the latest on the day of inclusion and before any study-related examination).

**6.2. EXCLUSION CRITERIA**

- Bilateral SCD diagnosed by high-resolution temporal bone CT, to avoid uncertainty in symptom attribution.
- Doubtful SCD diagnosis on high-resolution temporal bone CT (uncertainty regarding bone discontinuity, technical characteristics of the scan, or absence of specific reconstructions).
- Associated otologic or otoneurological pathology that could cause symptom impact similar to SCD: chronic otitis media, Eustachian tube dysfunction, otosclerosis and other ossicular anomalies with normal tympanum, definite Ménière’s disease, vestibular migraine.
- Patient under judicial protection or other protective regime (guardianship, curatorship).
- Pregnant women.

**6.3. FEASIBILITY AND RECRUITMENT MODALITIES**
Lyon, Bordeaux, and Toulouse are the three French centers with the most clinical experience in SCD management nationally and in related scientific publications.
The number of patients consulting for SCD exceeds 40/year in Lyon, Bordeaux, Montpellier, and Toulouse, and about 10/year at the University Hospital of La Réunion. Dr JF Vellin is the reference specialist in otology and otoneurology at CHU Saint-Denis, with specialized responsibilities for cochlear implants and complex otoneurological pathologies. Participants will be recruited during their consultations. General ENT correspondents at each center will be informed of the study and may refer eligible patients to their respective study center. Recruitment may also be facilitated by neuroradiology teams associated with each center, who make the radiological SCD diagnosis and can inform patients about the study.
Each of the 5 investigating centers is a nationally or internationally recognized expert center in otoneurology and functional hearing and balance assessments.

**7. STUDY TREATMENT/STRATEGY/PROCEDURES**

**7.1. EXPERIMENTAL TREATMENT/STRATEGY/PROCEDURE**
This study aims to correlate objective anomalies present in SCD at the electrophysiological or radiological level with SCD symptom severity. High-resolution temporal bone CT will have been performed prior to the study and will serve as an inclusion criterion. The electrophysiological assessments in this study are non-invasive and routinely used in otoneurology consultations. No adverse effects are expected beyond discomfort from the probe in the external auditory canal and/or loud sound delivery.

- **EcoG**: records cochlear and neural responses via an electrode near the tympanum to controlled auditory stimulation delivered via headphones.
- **WBT**: measures acoustic energy absorbed by the tympano-ossicular system using a tympanometry probe inserted in the external auditory canal delivering wideband sounds.
- **PEMV recording**: measures muscular responses to high-intensity auditory stimulation delivered via headphones using electrodes on the neck or lower eyelid.

**7.2. COMPARISON TREATMENT/STRATEGY/PROCEDURE**
Not applicable.

**7.3. PRODUCT CIRCUIT**
Not applicable.

**7.4. BLINDING**
Not applicable.

**8. ASSOCIATED TREATMENTS AND PROCEDURES**

**8.1. AUTHORIZED ASSOCIATED TREATMENTS/PROCEDURES**

**8.1.1. AUXILIARY MEDICATIONS**
Not applicable.

**8.1.2. OTHER TREATMENTS/PROCEDURES**
Patients may continue any treatments initiated prior to the start of the study.

**8.2. PROHIBITED ASSOCIATED TREATMENTS/PROCEDURES**
No therapeutic intervention for SCD is allowed during the participant’s participation in the study.

**9. STUDY CONDUCT**

**9.1. STUDY TIMELINE**

- Inclusion period duration: 18 months
- Participation duration per participant: 1 month
- Total study duration: 19 months

**9.2. PARTICIPANT FOLLOW-UP SUMMARY TABLE**

| **Stage** | **Pre-inclusion T0 (0 to -7 days)** | **Visit 1 Inclusion T0** | **Visit 2 Follow-up T15d (+/-5d)** | **Visit 3 End-of-study T30 (+/-5d)** |
| --- | --- | --- | --- | --- |
| Informed consent | ✓ |  |  |  |
| Clinical examination | ✓ |  |  |  |
| Temporal bone CT (S) | ✓ |  |  |  |
| VAS pulsatile tinnitus (R) |  | ✓ | ✓ | ✓ |
| VAS autophony (R) |  | ✓ | ✓ | ✓ |
| VAS sound-induced balance disorders (R) |  | ✓ | ✓ | ✓ |
| VAS pressure-induced balance disorders (R) |  | ✓ | ✓ | ✓ |
| Naert questionnaire (R) |  | ✓ | ✓ | ✓ |
| Electrocochleography (EcoG) (R) |  | ✓ | ✓ | ✓ |
| Ocular and cervical VEMP (R) |  | ✓ | ✓ | ✓ |
| Wideband tympanometry (WBT) (R) |  | ✓ | ✓ | ✓ |
| Evoked involuntary responses (EvI) |  | ✓ | ✓ | ✓ |

**9.3. PRE-INCLUSION/INCLUSION VISIT**

**9.3.1. CONSENT COLLECTION**
During the pre-inclusion/inclusion visit, the investigator informs the participant and answers all questions regarding the study’s objective, requirements, foreseeable risks, and expected benefits. The participant’s rights in the context of clinical research are also explained, and eligibility criteria are verified.

A copy of the information sheet and consent form is provided to the participant by the investigator. After this information session, the participant has time to consider participation. If the participant agrees, both the participant and the investigator clearly print their names, date, and sign the consent form. The consent form must be signed **before any clinical or paraclinical examination required by the study is performed**.

**9.3.2. VISIT PROCEDURE**
The pre-inclusion/inclusion visit is conducted by the investigator. Before any study-related examination, the investigator obtains free, informed, written consent from the participant (or legal representative, if applicable).

At the pre-inclusion visit, the previously performed temporal bone CT will be examined to formally confirm the SCD diagnosis.

At the inclusion visit, the following will be performed:

- Clinical examination and evaluation of cardinal symptoms (pulsatile tinnitus, autophony, sound-induced balance disorders, pressure-induced balance disorders)
- Naert questionnaire
- Electrophysiological measurements recorded twice on the healthy ear and twice on the affected ear:
  - Electrocochleography (EcoG)
  - Ocular and cervical VEMP
  - Wideband tympanometry (WBT)

**9.4. FOLLOW-UP VISITS**

**Visit 2 (15 days ± 5 days)**
During visit 2, performed on site by study staff:

- Clinical examination and evaluation of cardinal symptoms
- Naert questionnaire
- Electrophysiological measurements recorded twice on the healthy ear and twice on the affected ear:
  - Electrocochleography (EcoG)
  - Ocular and cervical VEMP
  - Wideband tympanometry (WBT)

**9.5. END-OF-STUDY VISIT**

**Visit 3 (30 days ± 5 days)**
During visit 3, performed on site by study staff:

- Clinical examination and evaluation of cardinal symptoms
- Naert questionnaire
- Electrophysiological measurements recorded twice on the healthy ear and twice on the affected ear:
  - Electrocochleography (EcoG)
  - Ocular and cervical VEMP
  - Wideband tympanometry (WBT)

**9.6. WITHDRAWAL RULES**

**9.6.1. PATIENT WITHDRAWAL FROM THE STUDY**
A participant may discontinue the study:

- By necessity, as decided by the investigator
- By personal decision: early withdrawal from the protocol

Regardless of the reason, patient care will remain the same as prior to study participation.

Participants may withdraw at any time without any specific responsibility or obligation. They are free to justify their decision. Data collected before withdrawal will be used unless the participant requests destruction.

Early withdrawals prior to visit 2 will be replaced.

**9.6.2. STUDY TERMINATION**
The study will normally end as planned in the protocol.
However, temporary suspension or early termination may occur due to:

- Significant violations of good clinical practice (GCP) compromising the primary objectives or participant safety
- Occurrence of local or global events (e.g., COVID-19 pandemic) impacting participant safety monitoring

**9.7. STUDY CONSTRAINTS AND PARTICIPANT COMPENSATION**
Study constraints are minimal (category 2, article L1121 of the French Public Health Code).
Participants may participate in another study concurrently.
No compensation is planned. Travel expenses to the study site will be covered by the study.

**9.8. COLLECTION OF BIOLOGICAL SAMPLES**
Not applicable.

**10. ADVERSE EVENTS / INCIDENT MANAGEMENT**
Adverse events, incidents, or side effects will be reported according to the applicable vigilance systems (healthcare, pharmacovigilance, hemovigilance, cosmetovigilance, etc.) in compliance with regulations.

Reporters must specify the patient is enrolled in a clinical trial and identify the specific study.

Investigators must immediately inform the sponsor of any patient safety concerns during the study.

No adverse events are expected under the study protocol.

Sponsor: CHU de TOULOUSE
Vigilance responsible: Dr. Pascale OLIVIER-ABBAL
Fax: +33 5 61 77 84 11
Email: vigilance.essaiscliniques@chu-toulouse.fr

**11. STATISTICAL ASPECTS**

**11.1. SAMPLE SIZE CALCULATION**
To evaluate reliability and validity of electrophysiological indicators, the sample size was defined to estimate reliability and validity parameters with sufficient precision.

100 patients with 6 repeated measurements will allow estimation of an intraclass correlation coefficient (ICC) > 0.80 with 95% CI width < 0.10.

Additionally, 100 patients allow the following precision for correlation values:

- 95% CI width < 0.30 for Pearson correlation ≥ 0.50
- 95% CI width < 0.40 for Pearson correlation = 0

**11.2. STATISTICAL METHODS**
Data will be described overall; outliers will be identified and corrected if possible. Missing data will be described for the population.

Inclusion/exclusion criteria, patient follow-up, and reasons for early withdrawal will be described, with a flow chart for inclusions and withdrawals.

Reliability of repeated measures will be evaluated first, as it allows further validity assessment:

- Electrophysiological indicators: intra-patient reproducibility (6 repeated measures), inter-visit (3 visits), intra-visit (2 measures per visit) using Bland-Altman plots (1999) with mean bias, 95% CI, and limits of agreement. Adaptation for more than 2 repeated measures will be used for inter-visit or intra-patient reliability (Jones et al., 2011). Intraclass correlation coefficients with 95% CI will also be calculated.
- The same reliability assessment applies to 3 repeated cardinal symptom measures.

If clinical and paraclinical measures are reliable, the validity of indicators will be analyzed using the mean of 6 repeated electrophysiological measures and the mean of 3 repeated clinical symptom measures.

- Convergent validity: correlations between electrophysiological indicators on the pathological (SCD) side and symptoms (presence, intensity, Naert questionnaire)
- Divergent validity: same analyses for the healthy side
- Correlations between electrophysiological indicators, anatomical-radiological measures, symptoms, and quality of life will be described to better understand construct validity.

If inter-visit reliability does not allow use of repeated-measure means, correlations may be estimated using mixed-effect models after standardization of variables. Psychometric properties of the French-translated Naert questionnaire will be assessed (inter-item correlations, item-total correlations, Cronbach’s alpha). Factor analysis will be performed.

Test-retest reproducibility of the Naert questionnaire will be evaluated by intraclass correlation coefficient and Bland-Altman indicators (3 repeated measures across 3 visits).

Unsupervised multivariate descriptive analysis (PCA, clustering) will define patient profiles based on electrophysiological, anatomical, and symptom characteristics. Supervised modeling (partial least squares regression) may also be applied to predict symptoms from multivariable indicators (+/- anatomical indicators).

**11.3. SAFETY ANALYSIS**
Not applicable.

**12. DATA AND SOURCE DOCUMENT MANAGEMENT**

**12.1. SOURCE DATA AND DOCUMENTS**
Source data consist of all information contained in original documents, or in certified copies of these documents, related to clinical examinations, observations, or other activities conducted in the context of research and necessary for reconstruction and evaluation of the study. Documents in which source data are recorded are called source documents.

In this study, source documents will consist of patients’ medical records, questionnaires completed at each visit, and reports of audiometric recordings.

**12.2. DATA COLLECTION INSTRUCTIONS**
All information required by the protocol must be recorded in the case report forms (CRFs), and an explanation must be provided for any missing data. Data must be collected as obtained and transcribed neatly and legibly into the CRFs.

Data will be collected in an electronic case report form (eCRF).

**12.3. DATA MANAGEMENT AND FLOW**
All information required by the protocol and necessary for evaluating study objectives will be recorded in the eCRF. Investigators or designated personnel are responsible for data entry into the eCRF. Investigators are responsible for ensuring data accuracy and signing the relevant pages.

The implementation and management of the eCRF will be handled by the USMR of CHU Toulouse. Data managers will be responsible for data cleaning for statistical analysis, and a data validation plan will be prepared.

Data managers will also code medical history and adverse events entered into the eCRF according to MedDRA, and concomitant treatments according to the WHO Drug dictionary. Versions of MedDRA and WHO Drug dictionaries will be defined at the start of the study by the sponsor. Data management software used is ENNOV CLINICAL.

**12.4. DATA CONFIDENTIALITY**
In accordance with applicable laws, all personnel with direct access to source data will take all necessary precautions to ensure confidentiality of information relating to experimental drugs, research participants, their identity, and study results. These personnel, like the investigators themselves, are bound by professional confidentiality.

During or after the study, data collected from participants and transmitted to the sponsor will be anonymized. Names and addresses must not be included.

Participants will be identified by a patient number corresponding to the center number followed by the patient’s inclusion order within that center.

The sponsor will ensure that each participant has given written consent for access to personal data strictly necessary for quality control of the study.

**12.5. STORAGE OF STUDY DOCUMENTS AND DATA**
The following documents will be archived by the investigator according to Good Clinical Practice, the French decree of 11 August 2008, and European regulations on medicinal products:

- For 15 years following the end of the study (for research not involving products mentioned in Article L.5311-1 of the Public Health Code):
  - Protocol and any amendments
  - Case report forms (copies)
  - Source documents of participants who have signed consent
  - All other study-related documents and correspondence
  - Original signed informed consent forms

These documents are the responsibility of the investigator during the regulatory retention period. No movement or destruction may occur without the sponsor’s agreement. After the retention period, the sponsor will be consulted regarding destruction. All data and documents may be subject to audit or inspection.

Study data will be accessible to authorized personnel from CHU Toulouse for two years after the last publication of results, and will be archived for 15 years after study completion in accordance with applicable regulations.

**12.6. DATA TRANSFER**
Data management is handled by [name of structure]. Conditions for transfer of all or part of the study database are determined by the sponsor and formalized in a written contract.

**13. MONITORING AND QUALITY ASSURANCE**

**13.1. DATA ACCESS**
Participation in the protocol implies that investigators will make available the documents and individual data strictly necessary for study monitoring, quality control, and audit to authorized personnel, in accordance with applicable laws and regulations.

**13.2. QUALITY CONTROL**
A clinical research associate (CRA) appointed by the sponsor will regularly visit each study site during study setup, periodically during the study, and at study completion. During these visits, and according to the risk-based monitoring plan (participant, logistics, impact, resources), the following will be reviewed:

- Informed consent
- Compliance with the protocol and defined procedures
- Data quality in CRFs: accuracy, missing data, consistency with source documents (medical records, appointment books, lab results, etc.)
- Management of any study products

Each visit will result in a written monitoring report.

**13.3. AUDIT AND INSPECTION**
An audit may be conducted at any time by sponsor-appointed personnel independent of study staff to verify participant safety, regulatory compliance, and data reliability.

An inspection may also be performed by a competent authority (e.g., ANSM in France, EMA for European trials). Audits and inspections can apply at any stage, from protocol development to publication and data archiving.

Investigators agree to comply with sponsor requirements for audits and competent authority inspections.

**14. ETHICAL AND REGULATORY CONSIDERATIONS**
The sponsor and investigators commit to conducting this study in accordance with French law n°2012-300 of 5 March 2012 on research involving humans, EU Regulation 536/2014 of 16 April 2014 on clinical trials of medicinal products, Good Clinical Practice (ICH v5, 1 December 2016 and 24 November 2006 decision), and the Declaration of Helsinki (full text: <http://www.wma.net>).

The study is conducted according to this protocol. Except in emergencies requiring specific therapeutic actions, investigators must follow the protocol precisely, particularly regarding informed consent and reporting and follow-up of serious adverse events.

This study received favorable opinion from the Ethics Committee (CPP) of [CPP name].

The sponsor has obtained civil liability insurance from [insurance company name] in accordance with the Public Health Code.

Data collected in this study are processed by [name of structure] in compliance with the French Data Protection Act (Law n°78-17 of 6 January 1978, amended by Law n°2018-493 of 20 June 2018) and the EU General Data Protection Regulation (GDPR, EU 2016/679).

This study follows the “Reference Methodology” (MR-001) under Article 54(5) of the French Data Protection Act and has CNIL approval. [Structure responsible for data processing] has signed a compliance commitment.

If applicable:

- This study is registered in the European database EudraCT/ID-RCB under n° [registration number]
- Registered on <http://clinicaltrials.gov/>
- Post-study storage of biological samples will be declared to the Ministry of Higher Education, Research and Innovation (and submitted to the CPP for approval if research purpose changes).

**PROTOCOL AMENDMENTS**
Substantial amendments (impacting participant protection, validity, study outcomes, product safety, or scientific interpretation) require a written amendment submitted to the sponsor and prior approval from the CPP, and if applicable, authorization from ANSM.

Non-substantial amendments (with no significant impact) are communicated to the CPP for information.

All amendments are validated by the sponsor and relevant study personnel before submission to the CPP and ANSM, and all investigators must be informed and comply with changes.

Any modification affecting participant care, benefits, risks, or constraints requires a new information sheet and consent form, following the same procedure as initial consent.

**15. FINAL REPORT**
Within one year of study completion or termination, a final report will be prepared and signed by the sponsor and investigator. The report will be made available to competent authorities. The sponsor will submit a summary of results to the CPP and, if applicable, to ANSM within one year of study completion.

**16. PUBLICATION RULES**

**16.1. SCIENTIFIC COMMUNICATION**
Data analysis is performed by [name of structure] and submitted to the sponsor, who will forward results to the CPP and competent authority.

Any written or oral communication of results requires prior agreement of the coordinating investigator and, if applicable, any study committee.

The coordinating/principal investigator commits to making all results available to the public, whether positive, negative, or inconclusive.

Primary results publications will mention the sponsor, all investigators who enrolled or followed participants, methodologists, biostatisticians, data managers, safety monitors, study committees, and, if applicable, funding sources. International standards for writing and publication (ICMJE, April 2010) will be followed.

**16.2. COMMUNICATION OF RESULTS TO PARTICIPANTS**
In accordance with French law n°2002-303 of 4 March 2002, participants will be informed, upon request, of the overall results of the study.

REfErences

-Adams ME, Kileny PR, Telian SA, El-Kashlan HK, Heidenreich KD, Mannarelli GR, Arts HA. Electrocochleography as a diagnostic and intraoperative adjunct in superior semicircular canal dehiscence syndrome. Otol Neurotol. 2011 Dec;32(9):1506-12.

-Bae JS, Lim HW, An YS, Park HJ. Acquired superior semicircular canal dehiscence confirmed by sequential CT scans. Otol Neurotol. 2013 Aug;34(6):e45-6.

-Berning AW, Arani K, Branstetter BF 4th. Prevalence of Superior Semicircular Canal Dehiscence on High-Resolution CT Imaging in Patients without Vestibular or Auditory Abnormalities. AJNR Am J Neuroradiol. 2019 Apr;40(4):709-712.

- Bland JM, Altman DG. Measuring agreement in method comparison studies. Stat Methods Med Res 1999;8:135. DOI: 10.1177/096228029900800204

-Chien WW, Janky K, Minor LB, Carey JP. Superior canal dehiscence size: multivariate assessment of clinical impact. Otol Neurotol. 2012 Jul;33(5):810-5.

-Eberhard KE, Chari DA, Nakajima HH, Klokker M, Cayé-Thomasen P, Lee DJ. Current Trends, Controversies, and Future Directions in the Evaluation and Management of Superior Canal Dehiscence Syndrome. Front Neurol. 2021 Apr 6;12:638574.

-Hall DA, Zaragoza Domingo S, Hamdache LZ, Manchaiah V, Thammaiah S, Evans C, Wong LLN; International Collegium of Rehabilitative Audiology and TINnitus Research NETwork. A good practice guide for translating and adapting hearing-related questionnaires for different languages and cultures. Int J Audiol. 2018 Mar;57(3):161-175.

-Hunter JB, Patel NS, O'Connell BP, Carlson ML, Shepard NT, McCaslin DL, Wanna GB. Cervical and Ocular VEMP Testing in Diagnosing Superior Semicircular Canal Dehiscence. Otolaryngol Head Neck Surg. 2017 May;156(5):917-923.

- Jones M, Dobson A, O’Brian S. A graphical method for assessing agreement with the mean between multiple observers using continuous measures. International Journal of Epidemiology 2011;40:1308–1313.

doi:10.1093/ije/dyr109

-Lookabaugh S, Kelly HR, Carter MS, Niesten ME, McKenna MJ, Curtin H, Lee DJ. Radiologic classification of superior canal dehiscence: implications for surgical repair. Otol Neurotol. 2015 Jan;36(1):118-25.

-Masaki Y. The prevalence of superior canal dehiscence syndrome as assessed by temporal bone computed tomography imaging. Acta Otolaryngol. 2011 Mar;131(3):258-62.

-Merchant GR, Röösli C, Niesten ME, Hamade MA, Lee DJ, McKinnon ML, Ulku CH, Rosowski JJ, Merchant SN, Nakajima HH. Power reflectance as a screening tool for the diagnosis of superior semicircular canal dehiscence. Otol Neurotol. 2015 Jan;36(1):172-7.

-Naert L, Ocak I, Griet M, Van de Berg R, Stultiens JJA, Van de Heyning P, Bisdorff A, Sharon JD, Ward BK, Van Rompaey V. Prospective Analysis of an Evidence-Based Symptom Set in Superior Canal Dehiscence Syndrome. Otol Neurotol. 2021 Feb 1;42(2):e186-e192.

-Nakajima HH, Pisano DV, Roosli C, Hamade MA, Merchant GR, Mahfoud L, Halpin CF, Rosowski JJ, Merchant SN. Comparison of ear-canal reflectance and umbo velocity in patients with conductive hearing loss: a preliminary study. Ear Hear. 2012 Jan-Feb;33(1):35-43.

-Nieto P, Gallois Y, Molinier CE, Deguine O, Marx M. Surgical treatments of superior semicircular canal dehiscence: A single‐centre experience in 63 cases. Laryngoscope Invest Otolaryngol. In press.

-Noij KS, Duarte MJ, Wong K, Cheng YS, Masud S, Herrmann BS, Curtin HD, Kanumuri VV, Guinan JJ Jr, Kozin ED, Tarabichi O, Jung DH, Lee DJ, Rauch SD. Toward Optimizing Cervical Vestibular Evoked Myogenic Potentials (cVEMP): Combining Air-Bone Gap and cVEMP Thresholds to Improve Diagnosis of Superior Canal Dehiscence. Otol Neurotol. 2018 Feb;39(2):212-220.

-Noij KS, Rauch SD. Vestibular Evoked Myogenic Potential (VEMP) Testing for Diagnosis of Superior Semicircular Canal Dehiscence. Front Neurol. 2020 Jul 21;11:695.

-Park JH, Lee SY, Song JJ, Choi BY, Koo JW. Electrocochleographic findings in superior canal dehiscence syndrome. Hear Res. 2015 May;323:61-7.

-Pfammatter A, Darrouzet V, Gärtner M, Somers T, Van Dinther J, Trabalzini F, Ayache D, Linder T. A superior semicircular canal dehiscence syndrome multicenter study: is there an association between size and symptoms? Otol Neurotol. 2010 Apr;31(3):447-54.

-Rodgers B, Lin J, Staecker H. Transmastoid resurfacing versus middle fossa plugging for repair of superior canal dehiscence: Comparison of techniques from a retrospective cohort. World J Otorhinolaryngol Head Neck Surg. 2016 Dec 4;2(3):161-167.

-Schwartz TR, Lindemann TL, Mongelluzzo G, Wackym PA, Gadre AK. Gray-Scale Inversion on High Resolution Computed Tomography of the Temporal Bone: An Observational Study. Ann Otol Rhinol Laryngol. 2021 Oct;130(10):1125-1131.

-Stimmer H, Hamann KF, Zeiter S, Naumann A, Rummeny EJ. Semicircular canal dehiscence in HR multislice computed tomography: distribution, frequency, and clinical relevance. Eur Arch Otorhinolaryngol. 2012 Feb;269(2):475-80.

-Streiner DL, Norman GR, Cairney J. Health measurement scales. A practical guide to their development and use. Fifth edition. Oxford University Press 2015.

-Remenschneider AK, Owoc M, Kozin ED, McKenna MJ, Lee DJ, Jung DH. Health Utility Improves After Surgery for Superior Canal Dehiscence Syndrome. Otol Neurotol. 2015 Dec;36(10):1695-701.

-Ward BK, Agrawal Y, Nguyen E, Della Santina CC, Limb CJ, Francis HW, Minor LB, Carey JP. Hearing outcomes after surgical plugging of the superior semicircular canal by a middle cranial fossa approach. Otol Neurotol. 2012 Oct;33(8):1386-91

-Ward BK, van de Berg R, van Rompaey V, Bisdorff A, Hullar TE, Welgampola MS, Carey JP. Superior semicircular canal dehiscence syndrome: Diagnostic criteria consensus document of the committee for the classification of vestibular disorders of the Bárány Society. J Vestib Res. 2021;31(3):131-141.

-Wenzel A, Ward BK, Ritzl EK, Gutierrez-Hernandez S, Della Santina CC, Minor LB, Carey JP. Intraoperative neuromonitoring for superior semicircular canal dehiscence and hearing outcomes. Otol Neurotol. 2015 Jan;36(1):139-45.

-Williamson RA, Vrabec JT, Coker NJ, Sandlin M. Coronal computed tomography prevalence of superior semicircular canal dehiscence. Otolaryngol Head Neck Surg. 2003 Nov;129(5):481-9.

-Ziylan F, Kinaci A, Beynon AJ, Kunst HP. A Comparison of Surgical Treatments for Superior Semicircular Canal Dehiscence: A Systematic Review. Otol Neurotol. 2017 Jan;38(1):1-10.

**Associate centers**

| *Nom* | *Prénom* | *Ville* | *Pays* | *Hôpital/Groupe Hospitalier**  *(*le cas échéant)* | *e-mail* | *Tél* | *Spécialité* |
| --- | --- | --- | --- | --- | --- | --- | --- |
| *DARROUZET* | *Vincent* | *Bordeaux* | *France* | *GH Pellegrin / CHU de Bordeaux* | *vincent.darrouzet@chu-bordeaux.fr* | *05 56 79 55 42* | *ORL* |
| *VENAIL* | *Frédéric* | *Montpellier* | *France* | *Hôpital Gui de Chauliac /CHU de Montpellier* | *f-venail@chu-montpellier.fr* | *04 67 33 68 90* | *ORL (Otologie et neurotologie)* |
| *VELLIN* | *Jean-François* | *La Réunion* | *France* | *CHU La Réunion GCS IRSAM* | *dr.jfvellin.orl@orange.fr* | *02 62 92 58 30* | *ORL* |
| *IONESCU* | *Eugen* | *Lyon* | *France* | *Hôpital Edouard Herriot/ HCL* | *eugen.ionescu@chu-lyon.fr* | *04 72 11 05 03* | *ORL (audiologie et explorations otoneurologiques)* |

**This version of the INTEX-DEHI protocol was translated into English using an artificial intelligence software to comply with PLOS ONE publication requirements.**
