# Supplementary material for: INTerest of electrophysiological and functional EXploration in the evaluation of symptomatic impact of superior semicircular canal DEHIscence syndrome (INTEX-DEHI study): Study protocol for a reliability and validity study
Source: PLoS One. 2025 Sep 18;20(9):e0331763. doi: 10.1371/journal.pone.0331763 (PMC12445554; doi:10.1371/journal.pone.0331763)
Supplement: Attachment — Submitted filename: Response to PLOS One.pdf [file pone.0331763.s005.pdf]

Dear, members of the Editorial Board, and Reviewer,

We thank you for giving us the opportunity to improve our manuscript and for the elements provided for our submission.

We remain, of course, at your disposal for any necessary modifications.

We believe we have addressed all your requests.

Kind regards.

LEGOIS Quentin and co-authors

1. We note that you have indicated that you received funding for your study. If you received peer reviewed external funding, your submission may be considered for expedited editorial assessment. To be considered for expedited review, please upload the Funding letter you received, and English translation, as an 'Other' file. Please also provide proof that the funder administered independent peer review of your protocol. Examples of evidence include: a link to a description of the funder's peer-review process; a letter from the funding agency confirming the independent peer review process; a list of the names and credentials of the peer reviewers (if available). Please note that we will not consider the following types of funding for expedited peer review: Funding from an author's institution, or any other non-independent source; Funding from a commercial source; Funding that was not granted for this specific project (e.g. general PhD funding or fellowship). If you are unable to provide proof of peer-reviewed external funding for your study, please note this in your cover letter to avoid delays in processing your manuscript. More information related to the relevant journal policies is available from: [https://journals.plos.org/plosone/s/submission-guidelines#loc-study-protocols].

Indeed, we received public and independent external funding from the GIRCI-SOHO (Interregional Group for Clinical Research and Innovation – South-West and Overseas Hospital Network). Our project was indeed peer-reviewed. We are pleased to learn that our submission may be considered for expedited editorial assessment.

In the file "Other 1," you will find a letter issued by the Directorate of Research and Innovation, which managed the call for projects. This letter confirms the funding received and also explains how it has been and will be disbursed.

At the following internet link, you will find the results of this call for projects (PHRCI 2022), for which we were awarded third place with funding in the amount of €289,772.00: PHRC Interrégional 2021 | GIRCI SOHO .

You will also find on this website, which we remind you is public and independent, a description and evidence that the funding body conducted an independent peer review of our research project.

Modalités de dépôt et de sélection

Dépôt des LI : les LI seront transmises par les DRCI à la coordination du GIRCI le **mardi 30 novembre 2021 23h59** au plus tard (se rapprocher de sa DRCI pour connaître la deadline précise au niveau local).
Expertise des LI par 2 experts, 1 scientifique, 1 méthodologiste, issus d'un établissement non impliqué au sein du GIRCI ; la grille d'évaluation est disponible en bas de page.
Sélection des LI : **vendredi 21 janvier 2022 en webconférence**
Pour les LI sélectionnées, dépôt des dossiers complets : se rapprocher de la DRCI de proximité pour connaître la date
Phase d'expertises : 2 expertises scientifiques et 1 méthodologique ; la grille d'évaluation est disponible en bas de page.
Conseil scientifique : **début juillet 2022.**

English translation :

Submission and Selection Procedures
Submission of LOIs: LOIs will be sent by the DRCIs to the GIRCI coordination team no later than Tuesday, November 30, 2021, at 11:59 p.m. (please contact your local DRCI to know the exact local deadline).
LOI review: Each LOI will be evaluated by 2 experts — 1 scientific expert and 1 methodological expert — from an institution not involved in the GIRCI; the evaluation form is available at the bottom of the page.
LOI selection: Friday, January 21, 2022, via web conference.
For the selected LOIs, submission of full proposals: please contact your local DRCI to know the date.
Review phase: 2 scientific reviews and 1 methodological review; the evaluation form is available at the bottom of the page.
Scientific Council: early July 2022.

2. Please note that Study Protocols must relate to a research study that has not yet generated results. Studies that have completed either recruitment or data collection will not be considered. For further information, please see https://journals.plos.org/plosone/s/submission-guidelines#loc-study-protocols. Please ensure that your Methods section reports the status and timeline of the study, including an estimate of when A) participant recruitment will be completed (if applicable), B) data collection will be completed and C) results are expected. If any of these stages have already been completed, this should be indicated in the Methods section.

You are correct, we have specified this in the Materials and Methods section. The manuscript file will be uploaded with the new submission, and you will find the changes made on page 6. These changes are highlighted in yellow and are presented below: ***"The study began on April 29, 2024, is still ongoing, and is planned for a duration of 24 months. Patient recruitment will therefore be completed on April 29, 2026, and data collection will be completed on May 29, 2026 (one month after the last inclusion). The data will then be processed to extract the results, which will be available within three months of that date."***

3. Please note that you must upload a completed SPIRIT schedule as Fig 1 of your manuscript. Blank copies of this document and information regarding SPIRIT schedules can be found here: http://www.spirit-statement.org/schedule-of-enrolment-interventions-and-assessments/" http://www.spirit-statement.org/schedule-of-enrolment-interventions-and-assessments/

At your request, we have created the SPIRIT schedule as Figure 1. This document will be included in the submission as Fig. 1. The place where it should be positioned in the manuscript has been added and highlighted in yellow. We have also renamed the previous Figure 1 to Figure 2. This figure will also be included at the time of submission in the document titled Fig. 2.

4. Our author guidelines for Clinical Trial Study Protocols require that you upload a SPIRIT checklist as Supporting Information. Information about the SPIRIT guidance and blank checklists can be found here: https://www.spirit-statement.org/.

Indeed, we had completed the SPIRIT checklist but not in the format required by your journal. We have adopted your format and completed the document accordingly. We will attach it with our next submission under the name “S1 Additional file.”

5. We note that you did not include a copy of the original, (signed or stamped) dated IRB/research ethics committee approval letter or waiver for all study sites as an ‘Other’ file. In keeping with our submission guidelines, please upload the approval letter or waiver as an ‘Other’ file. More information related to the relevant journal policies is available from: [https://journals.plos.org/plosone/s/submission-guidelines#loc-guidelines-for-specific-study-types]

The document we sent you was indeed neither signed nor dated. We therefore contacted the ethics committee, which informed us that this was not part of their protocol. However, they agreed to sign it electronically. We are attaching this signed document in the file “Other 2.” The translation of this document is in the file “Other 3. We are also including, in the file “Other 4,” a letter from the Directorate of Research and Innovation of our institution confirming that we received a favorable opinion from the ethics committee on December 10, 2023.
